# The MYC-dependent lncRNA MB3 inhibits apoptosis in Group 3 Medulloblastoma by regulating the TGF-β pathway via HMGN5

**DOI:** 10.1038/s41419-025-08097-8

**Published:** 2025-11-06

**Authors:** Alessia Grandioso, Paolo Tollis, Francesca Romana Pellegrini, Elisabetta Falvo, Alessandro Palma, Francesco Migliaccio, Alessandro Belvedere, Jessica Rea, Giada Tisci, Annamaria Carissimo, Irene Bozzoni, Daniela Trisciuoglio, Monica Ballarino, Pierpaolo Ceci, Pietro Laneve

**Affiliations:** 1 Department of Biology and Biotechnologies Charles Darwin, Sapienza University of Rome, Rome, Italy; 2 Institute of Molecular Biology and Pathology, National Research Council of Italy, Rome, Italy; 3 Center for Life Nano- & Neuro-Science, Fondazione Istituto Italiano di Tecnologia, Rome, Italy; 4 Department of Electrical Engineering and Information Technology, University Federico II, Naples, Italy; 5 Institute for Applied Mathematics “Mauro Picone”, National Research Council of Italy, Naples, Italy; 6 Department of Biochemical Sciences, Sapienza University of Rome, Rome, Italy

**Keywords:** Oncogenes, Long non-coding RNAs, RNA metabolism

## Abstract

Group 3 (G3) is one of the most common and aggressive subtypes of the paediatric cerebellar tumour Medulloblastoma (MB), primarily driven by the MYC oncogene. The challenging targeting of MYC, coupled with gaps in understanding G3 MB molecular bases, has hindered the development of targeted therapies. The unconventional oncogenic roles of long noncoding RNAs (lncRNAs) offer opportunities to address this complexity, to provide insights and to identify novel targets. Using -omics approaches and molecular/cellular assays, we elucidate the mode-of-action of lncMB3, a MYC-dependent, anti-apoptotic lncRNA in G3 MB. LncMB3 regulates the TGF-β pathway, critically altered in G3 medulloblastomagenesis, via direct binding and translational inhibition of the mRNA for the epigenetic factor HMGN5. This regulatory axis affects apoptosis through photoreceptor lineage genes, including the G3 driver OTX2. The synergistic effects between lncMB3 targeting and cisplatin treatment underscore the relevance of this network. Additionally, we propose novel ferritin-based nanocarriers for the efficient delivery of antisense oligonucleotides against lncMB3. LncMB3 crucially links MYC amplification and apoptosis inhibition through a circuit involving RNA-based mechanisms, G3 MB key determinants and underexplored factors. This integrated framework deepens the understanding of G3 MB landscape and supports the potential for translating lncRNA research into future applications.

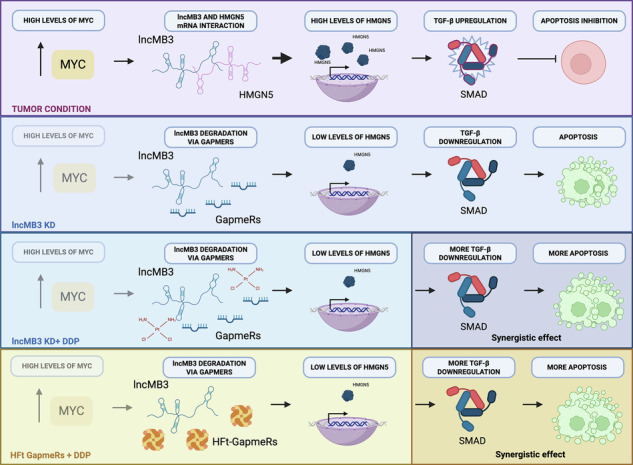

## Introduction

Medulloblastoma (MB) is the most prevalent malignant paediatric central nervous system tumour, accounting for ∼20% of all childhood brain cancers [1, 2]. Although all MBs arise in the cerebellum, share histomorphological features, and exhibit embryonic cerebellar lineage signatures [3], they show high heterogeneity. MB is classified into 4 subtypes, each with distinct biological, clinical and therapeutical implications [4]: Wingless (WNT), Sonic Hedgehog (SHH), Group 3 (G3) and Group 4 (G4). G3 and G4, together, account for 60% of MB cases and are the most aggressive, posing treatment challenges due to poor biochemical characterisation. Deciphering the molecular mechanisms of these subgroups is critical for advancing precision medicine. G3 MB presents a dismal prognosis, often with metastases at diagnosis and high recurrence rates [2], primarily driven by the oncogenic transcription factor (TF) MYC, characterised by gene copy number amplification or elevated expression [5]. Despite challenges in addressing canonical MYC circuits, targeting MYC and related pathways/complexes is essential in G3 MB management [6].

The discovery of novel classes of noncoding RNAs (ncRNAs) broadens our understanding of functional genome outputs [7], offering new therapeutic avenues for cancer. Among ncRNAs, the versatile long noncoding RNAs (lncRNAs) have emerged as key regulators of cancer hallmarks [8], showing promise as biomarkers and targets due to their cell-specific expression and regulatory roles. Over the years, we have explored ncRNAs in various nervous system cancer models, including MB [9]. Recently, we discovered MYC-dependent lncRNAs in G3 MB, three of them potentially acting as oncogenes [10], including *lncMB3*. Here, we functionally analyse *lncMB3*, which is overexpressed in tumour conditions and exerts an anti-apoptotic action. We show that *lncMB3* modulates the TGF-β pathway, significantly altered in G3 MB [11], through a functional interaction with the mRNA for the epigenetic factor HMGN5, whose protein levels are upregulated and impact on a subset of TGF-β pathway genes also affected by *lncMB3* targeting. This regulatory axis influences apoptosis through OTX2, another crucial G3 MB driver gene [12], and its downstream cascade. The relevance of this circuit is underscored by the synergistic effects observed when *lncMB3* targeting is paired with cisplatin (DDP) administration in G3 MB cells. Lastly, we demonstrate that recombinant human ferritin-based (HFt) nanovectors, a promising platform for drug delivery in diseased conditions [13], effectively carry antisense oligonucleotides (ASOs) targeting *lncMB3* in G3 MB cells.

## Results

### Identification of *lncMB3*-dependent transcriptome in G3 MB cells

To uncover the molecular network downstream of *lncMB3* in MB, we conducted transcriptome analysis post-knockdown (KD) in the MYC-amplified D283 Med cell line, where the lncRNA is highly expressed (Fig. S1A) and was first identified [10]. GTEx data (https://gtexportal.org/home/) show minimal expression of *lncMB3* in healthy tissues (Fig. S1B). KD reduced *lncMB3* expression by ∼70% (Fig. S2A), 72 h after multi-pulse transfections of a Locked Nucleic Acid (LNA)-based ASO [14], named GapmeR #1, targeting the lncRNA 5′ region. Transcriptome data analysis showed robust gene detection (Fig. S2B, left panel), accurate transcriptome clustering (Fig. S2B, right panels) and consistency of *lncMB3* KD normalised read counts with previous qRT-PCR analyses (Dataset 1).

**Fig. 1:**
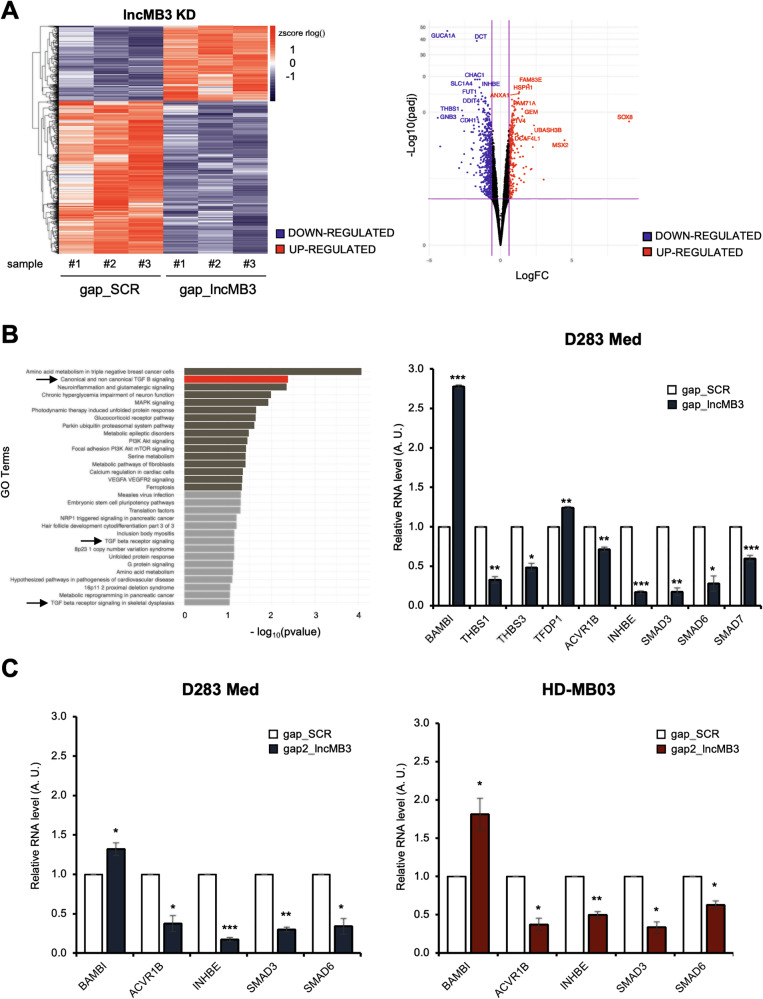
Differential gene expression analysis upon *lncMB3* KD in G3 MB cells. **A** Left Panel: heatmap showing the relative levels of DEGs according to RNA-Seq analyses, along with genes hierarchical clustering (3 biological replicates for each condition). Right Panel: volcano plot showing DEG distribution. Genes were plotted based on statistical significance −log_10_(FDR) and differential expression log_2_(FC). Downregulated genes (logFC < −0.6) are indicated by blue dots; upregulated genes (logFC > 0.6) by red dots. Invariant genes are indicated in black. **B** Left Panel: GO (wikipathways) showing the distribution in clusters of the DEGs in gap_*lncMB3 vs* gap_SCR-treated D283 Med cells, according to RNA-seq data. Categories are listed considering –log_10_(pvalue) and TGF-β-related ones are pointed by arrows. Right Panel: qRT-PCR validation analysis of TGF-β pathway genes in D283 Med cells treated for 72 h with gap_SCR or gap_*lncMB3* (GapmeR #1). Expression levels were compared to gap_SCR sample as control, set as 1. Data (means ± SEM) are expressed in arbitrary units (A.U.) and are relative to *GAPDH* mRNA levels. *N* = 3, * *p* ≤ 0.05, ** *p* ≤ 0.01, *** *p* ≤ 0.001 (two-tailed Student’s *t*-test). **C** Left Panel: qRT-PCR analysis of TGF-β pathway genes in D283 Med cells treated for 72 h with gap_SCR or gap2_*lncMB3* (GapmeR #2). Right Panel: HD-MB03 cells treated as above (GapmeR #2). Expression levels were compared to gap_SCR sample as control, set as 1. Data (means ± SEM) are expressed in arbitrary units and are relative to *GAPDH* mRNA levels. *N* = 3 to 5, * *p* ≤ 0.05, ** *p* ≤ 0.01, *** *p* ≤ 0.001 (two-tailed Student’s *t*-test).

RNA-Seq analysis (Dataset 1) detected 14655 transcripts expressed in at least one sample and identified 2995 differentially expressed genes (DEGs) between *lncMB3* KD and control (SCR), with FDR < 0.05 (Fig. 1A, left panel). Among them, 1395 were downregulated and 1600 upregulated following *lncMB3* silencing (Fig. 1A, right panel, blue and red dots). To technically validate the transcriptome profile and confirm RNA-seq expression trends across a range of statistical confidence, 14 of these genes were selected, with padj ranging from 0.001 to 0.05, irrespective of their biological function (Fig. S2C and Dataset 1).

To functionally explore the transcriptomic landscape, DEGs were filtered by quantitative criteria, such as fold change (FC) robustness, statistical significance and expression levels (Fig. S2D). Thirteen RNAs regulated by *lncMB3* were identified (12 protein-coding and 1 lncRNA): 7 significantly downregulated and 6 upregulated (Dataset 2). qRT-PCR validated RNA-Seq data for the selected DEGs, displaying statistical significance (10 out of 13 genes) or discernible variance trends (Fig. S3A).

Throughout this study, we validated by qRT-PCR, achieving statistical significance, 37 out of 40 genes identified through RNA-Seq, yielding a validation rate of approximately 93%.

The top DEG identified was *INHBE*, a component of the TGF-β pathway [15], associated with G3 MB pathogenesis [11].

Gene Ontology (GO) analysis of DEGs highlighted the full set of functional gene categories affected by *lncMB3* depletion (Fig. 1B, left panel). Among the top ontological annotations, GO revealed pathways such as “PIK3-Akt signalling”, associated to chemosensitivity in various cancers, including MB [16], and “MAPK signalling”, potentially reflecting apoptosis-induced DNA damage following *lncMB3* KD. Focus remained on the TGF-β pathway, the second most prominent gene category, deregulated in G3 compared to other MB subgroups. It appeared in both the up and downregulated gene sets (Fig. S3B), suggesting deregulation of both activatory and inhibitory elements in the same pathway. Gene Set Enrichment Analysis (GSEA) confirms the significant impact of *lncMB3* KD on the TGF-β pathway in G3 MB cells (Fig. S3C).

### *LncMB3* regulates the TGF-β pathway in G3 MB

Aberrant TGF-β signalling is involved in the development of numerous diseases, including cancer [17]. In G3 MB, its dysregulation is believed to drive processes such as cell proliferation, differentiation and survival [11].

The importance of the TGF-β cascade in our system and conditions was further highlighted by enriching the ontological category with additional DEGs involved in the pathway. We identified a set of 9 genes, including TGF-β/Activin signalling ligands (*INHBE*, [15]; *THBS1*, *THBS3*, [18]), receptors (*ACVR1B*,[19]; *BAMBI*, [20]), effectors (*SMAD3*, *SMAD6*, *SMAD7*, [19]) and downstream regulators (*TFDP1*, [21]) (Fig. S3D). Upon *lncMB3* KD, qRT-PCR showed repression of genes with activatory functions in the pathway (*THBS1*, *THBS3*, *INHBE*, *ACVR1B*, *SMAD3*, *SMAD6*, *SMAD7*), with reductions ranging from 20% to 80% (Fig. 1B, right panel). Conversely, inhibitory components (*BAMBI* and *TFDP1*) were upregulated (1.5- to threefold, Fig. 1B, right panel), suggesting a coherent and coordinated contribution of both positive and negative effects leading to overall downregulation of the TGF-β/Activin branch of the network. To ensure specificity of targeting, we employed a second GapmeR (GapmeR #2), which recognises the 3′ end of *lncMB3* (Fig. S4A), achieving 70% transcript depletion in D283 Med cells (Fig. S4B). In HD-MB03, another G3 MB cell line where MYC and *lncMB3* are upregulated (Fig. S1A) and that we used to further corroborate our findings, GapmeR #2 reduced *lncMB3* levels by 50% (Fig. S4B). Validations showed similar expression changes across all the tested conditions for 5 TGF-β network genes: *BAMBI*, *ACVR1B*, *INHBE*, *SMAD3* and *SMAD6* (Fig. 1C). Since GapmeR #1 targets the 5′ end of *lncMB3* and results in a more efficient downregulation (Fig. S4B), it was preferred for subsequent experiments. To corroborate *lncMB3* as a regulator of the TGF-β pathway, we analysed the publicly available transcriptomic dataset GSE164677, comparing 59 patient-derived samples across all MB subgroups and 4 controls. We stratified MB samples into 3 groups: healthy controls (Cereb), G3 MB patients with high *lncMB3* expression (High), and G3 MB patients with low/negative *lncMB3* expression (Low). As expected, and despite the limited sample size, *MYC* expression—used here as a positive control—appears to associate with *lncMB3* levels in this dataset (Fig. S4C). More specifically, results suggest a correlation trend between the TGF-β genes *ACVR1B*, *INHBE*, *SMAD3* and *SMAD6* and *lncMB3* (Fig. S4C), in term of increased median expression in the *lncMB3*-High group compared to normal tissue, and a decrease in the *lncMB3*-Low group. As a negative control, we examined the expression of genes excluded from the *lncMB3* core target list due to inconsistent regulation with one of the two GapmeRs or in one cell line (Fig. 1C), and we appreciated a lack of consistent patterns in patient samples (Fig. S4D). To further confirm the relevance of the genes of interest, we compared the distribution of a composite differential expression score (“–log10(padj) × log2FoldChange”) between validated and non-validated target gene groups in High *vs* Low (including healthy cerebella) *lncMB3* samples. The Wilcoxon rank-sum test revealed a significant difference between them (*p* = 0.029), indicating that, overall, *lncMB3* targets show consistent expression changes with *lncMB3* in G3 MB samples (Fig. S4E). Overall, in vitro and in vivo RNA analyses point to a role for *lncMB3* in the regulation of the TGF-β pathway components in G3 MB.

### Insights into apoptosis regulation by *lncMB3*

In the developing nervous system, a relevant TGF-β cascade target is the brain-specific TF OTX2, known as a G3 MB-enriched oncogene [12], upregulated in both D283 Med and HD-MB03 cells (Fig. S1A). During retinal development, *OTX2* occupies a pivotal position in a photoreceptor gene hierarchy, including basic leucine zipper TFs *NRL* and *CRX* [22], both overexpressed in G3 MB, where they promote cancer survival via the anti-apoptotic factor *BCL2L1* ([23] and Fig. S3D).

**Fig. 2:**
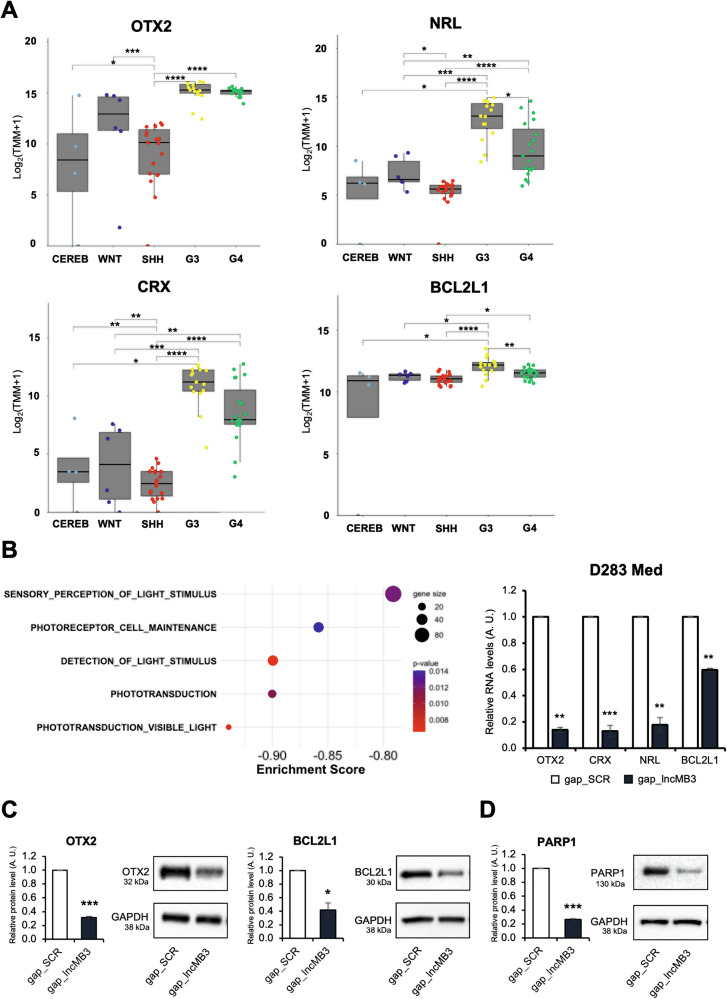
Expression of *OTX2*, *NRL*, *CRX* and *BCL2L1* in MB. **A** *OTX2*, *NRL*, *CRX* and *BCL2L1* mRNA expression in *N* = 4 healthy cerebella (CEREB) and *N* = 59 primary MB subgroup samples, according to the transcriptomic dataset GSE164677. Results are expressed in log_2_(TMM + 1). * *p* ≤ 0.05, ** *p* ≤ 0.01, *** *p* ≤ 0.001 **** *p* ≤ 0.0001 (two-tailed Student’s *t*-test). **B** Left Panel: GSEA biological process (BP) domains performed on deregulated genes. Categories are listed considering enrichment score, gene size and *p*-value. Right Panel: qRT-PCR analysis of *OTX2*, *NRL*, *CRX* and *BCL2L1* in D283 Med cells treated for 72 h with gap_SCR or gap_*lncMB3* (GapmeR #1). Expression levels were compared to gap_SCR sample as control, set as 1. Data (means ± SEM) are expressed in arbitrary units and are relative to *GAPDH* mRNA levels. *N* = 3, ** *p* ≤ 0.01, *** *p* ≤ 0.001 (two-tailed Student’s *t*-test). **C** Western blot analysis of OTX2 (left panel) and BCL2L1 (right panel) protein levels in D283 Med cells treated for 72 h with gap_SCR or gap_*lncMB3* (GapmeR #1). Normalisations are relative to GAPDH protein levels. *N* = 3, * *p* ≤ 0.05, *** *p* ≤ 0.001, (two-tailed Student’s *t*-test). **D** Western blot analysis of PARP1 protein levels in D283 Med cells treated for 72 h with gap_SCR or gap_*lncMB3* (GapmeR #1). Details as in (**C**).

To evaluate these genes in primary MB tumours, we again queried the dataset GSE164677. *NRL*, *CRX* and *BCL2L1* expression was significantly upregulated in G3 compared to healthy tissues or other MB subgroups. Although *OTX2* upregulation in G3 MB *vs* cerebellum was not statistically significant, it showed a rising trend (Fig. 2A), which was, however, insufficient to result in a linear correlation with *lncMB3* expression (data not shown), possibly due to *OTX2* indirect and context-dependent regulation by *lncMB3*. Nevertheless, the photoreceptor gene program appeared in several downregulated annotations in *lncMB3* KD-dependent GSEA (Fig. 2B, left panel).

**Fig. 3:**
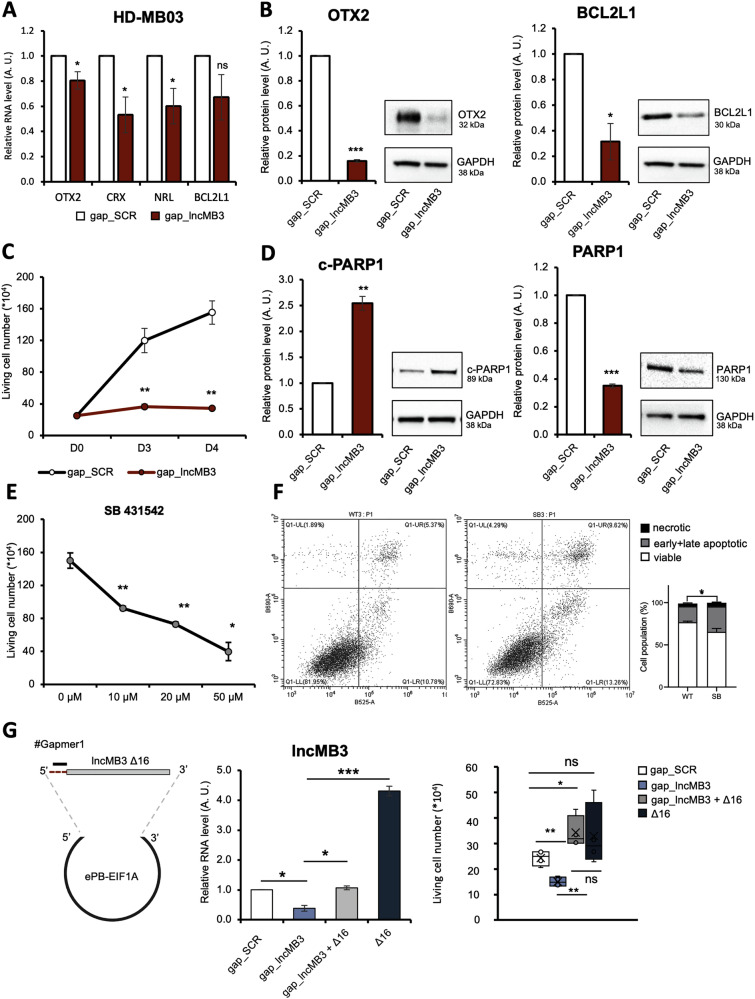
Analysis of TGF-β pathway and apoptosis in G3 MB cells. **A** qRT-PCR analysis of *OTX2*, *NRL*, *CRX* and *BCL2L1* in HD-MB03 cells treated for 72 h with gap_SCR or gap_*lncMB3* (GapmeR #1). Expression levels were compared to gap_SCR sample as control, set as 1. Data (means ± SEM) are expressed in arbitrary units and are relative to *GAPDH* mRNA levels. *N* = 3, * *p* ≤ 0.01 (two-tailed Student’s *t*-test). **B** Western blot analysis of OTX2 (left panel) and BCL2L1 (right panel) protein levels in HD-MB03 cells treated for 72 h with gap_SCR or gap_*lncMB3* (GapmeR #1). Normalisations were performed relative to GAPDH protein levels. *N* = 3, * *p* ≤ 0.05, *** *p* ≤ 0.001, (two-tailed Student’s *t*-test). **C** Time course analysis of the number of viable HD-MB03 cells depleted for *lncMB3* (gap_*lncMB3*). Scramble-transfected cells (gap_SCR) were used as control. Cells were transfected at day 0 (D0) and counted at each timepoint. Data (means ± SEM) are expressed as the number of viable cells, counted by an automated cell counter. *N* = 4, ** *p* ≤ 0.01, (two-tailed Student’s *t*-test). **D** Western blot analysis of c-PARP1 (left panel) and PARP1 (right panel) protein levels in HD-MB03 cells treated for 72 h with gap_SCR or gap_*lncMB3* (GapmeR #1). *N* = 3, ** *p* ≤ 0.01, *** *p* ≤ 0.001. Details as in (**B**). **E** Dose-response analysis of the number of viable D283 Med cells after TGF-β inhibition. Control cells were treated with vehicle (DMSO). Cell counts started 24 h after treatment. Data (means ± SEM) are expressed as the number of viable cells, counted by an automated cell counter. *N* = 4, * *p* ≤ 0.05, ** *p* ≤ 0.01 (two-tailed Student’s *t*-test). **F** Left Panel: representative flow cytometry analysis of propidium iodide- and Annexin V-stained D283 Med cells upon TGF-β inhibition. X-axis Annexin V-FITCH staining; Y-axis: PI staining. Right Panel: quantification of the fractions (expressed as percentage of total cell number) of viable (Annexin V−/PI−), early apoptotic (Annexin V+/PI−), late apoptotic (Annexin V+/PI+) and necrotic (Annexin V−/PI+) cells. For early+late apoptotic cells, *N* = 3, * *p* ≤ 0.05 (two-way ANOVA). **G** Left panel: schematic representation of the construct overexpressing *lncMB3* Δ16. Middle panel: qRT-PCR analysis of *lncMB3* expression in D283 Med cells transfected with gap_SCR, gap_*lncMB3* or gap_*lncMB3* + Δ16 construct, Δ16 construct alone. *N* = 3, * *p* ≤ 0.05, *** *p* ≤ 0.001. Right panel: number of viable D283 Med cells upon gap_*lncMB3* transfection (*lncMB3* KD) or Δ16 co-expression, or Δ16 expression alone, compared to gap_SCR condition. Normalisation was performed on *lncMB3* KD condition. *N* = 5, * *p* ≤ 0.05, ** *p* ≤ 0.01 (two-tailed Student’s *t*-test).

*OTX2*, *NRL*, *CRX* and *BCL2L1* RNAs were downregulated in D283 Med cells after *lncMB3* depletion (Fig. 2B, right panel). Protein downregulation was also assessed for OTX2 and BCL2L1 (Fig. 2C), as well as for PARP-1 (Fig. 2D) [24], whose cleavage product (C-PARP) is an apoptosis hallmark. Further confirmation of the lncRNA involvement in this axis was obtained in HD-MB03 cells using GapmeR #1, with *lncMB3* depletion leading to (i) reduced RNA and protein levels for OTX2, NRL, CRX and BCL2L1 (Fig. 3A, B); (ii) decreased cell viability (Fig. 3C); and (iii) corresponding modulation of C-PARP and PARP-1 levels (Fig. 3D).

Consistent with *lncMB3* regulation by MYC [10], RNA alterations seen with *lncMB3* KD were also observed in MYC-inhibited D283 Med cells *vs* untreated cells (Dataset 3). To further link *lncMB3* and the TGF-β pathway within the same regulatory network, we used a TGF-β Receptor Kinase inhibitor to block Smad2/3 phosphorylation. After 48 h-treatment, we observed a dose-dependent reduction in the number of viable D283 Med cells, which were prioritised in all drug assays (Fig. 3E), correlated with increased apoptosis via Annexin V assay (Fig. 3F). These findings show that direct TGF-β cascade inhibition produced effects converging with *lncMB3* silencing.

To definitively confirm *lncMB3* role in counteracting apoptosis, we performed a rescue assay. A plasmid carrying the lncRNA sequence lacking its first 16 nucleotides (Δ16), necessary for GapmeR #1 recognition, was generated (Fig. 3G, left panel). When ectopically expressed in D283 Med cells along with the ASO, the mutant lncRNA (Fig. 3G, middle panel) restored cell viability compared to cells transfected with GapmeR #1 alone (Fig. 3G, right panel). No consequences were observed when Δ16 was overexpressed alone, suggesting a saturation of the baseline cell viability rate that masked any additional pro-survival effect.

These findings reconstruct the molecular network initiated by *MYC* amplification and involving *lncMB3* as a trans-acting non-coding RNA that suppresses apoptosis in G3 MB.

### Insights into the RNA interactome of *lncMB3*

The lack of coding potential in lncRNAs necessitates the identification of their molecular partners as a strategy to understand their mechanisms of action [25]. To investigate how *lncMB3* modulates TGF-β and apoptotic pathway genes through such interactions, we adopted a stepwise approach guided by hypotheses of increasing complexity, with a particular focus on RNA–RNA interactions, given their emerging significance in molecular oncology [26, 27] and their suitability for experimental validation.

**Fig. 4:**
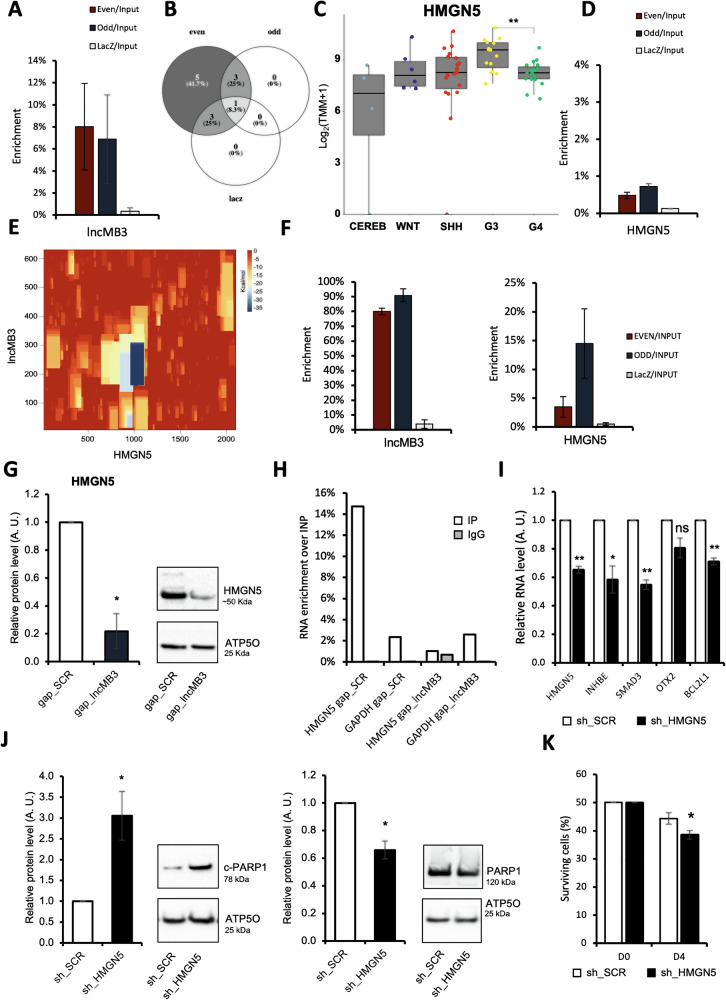
Analysis of *lncMB3*/*HMGN5* mRNA interaction. **A** qRT-PCR analysis of RNA enrichment in EVEN, ODD and LacZ fractions over Input, from native *lncMB3* RNA pull-down experiments in D283 Med cell extracts. Data expressed as percentage of Input, *N* = 2. **B** Venn diagram showing intersections of mRNAs retrieved and sequenced from two independent native *lncMB3* pull-down fractions (EVEN, ODD, LacZ). **C** *HMGN5* mRNA expression in *N* = 4 healthy cerebella (CEREB) and *N* = 59 primary MB subgroup samples, according to the transcriptomic dataset GSE164677. Results are expressed in log_2_(TMM + 1). * *p* ≤ 0.05, ** *p* ≤ 0.01, *** *p* ≤ 0.001, **** *p* ≤ 0.0001 (two-tailed Student’s *t*-test). **D** qRT-PCR analysis of *HMGN5* mRNA enrichment in native *lncMB3* RNA pull-down fractions. Details as in (**A**). Data are expressed as percentage of Input, *N* = 2. **E** IntaRNA energy map representing the predicted stability of the RNA-RNA interaction between *lncMB3* and *HMGN5* mRNA. mRNA and lncRNA sequences are positioned along the x- and y-axes, respectively. The free energy of predicted intramolecular pairs ranges from red (higher energy, unstable pairing) to blue (minimal energy, stable pairing). **F** qRT-PCR analysis of *lncMB3* (left panel) and *HMGN5* mRNA (right panel) enrichment in EVEN, ODD and LacZ fractions over Input, from AMT-crosslinked *lncMB3* RNA pull-down experiment in D283 Med cells. Data expressed as percentage of Input, *N* = 3. **G** Western blot analysis of HMGN5 protein levels in D283 Med cells treated for 72 h with gap_SCR or gap_*lncMB3* (GapmeR #1). Normalisations were performed relative to GAPDH protein levels. *N* = 3, * *p* ≤ 0.05, (two-tailed Student’s *t*-test). **H** qRT-PCR analysis of *HMGN5* and *GAPDH* upon RPL22-FLAG RIP assay in gap_SCR and gap_*lncMB3* (GapmeR #1) conditions. Analysis of RNA enrichment in IP and IgG fractions over Input. *N* = 1. Data expressed as percentage of Input. **I** qRT-PCR analysis of *HMGN5*, *INHBE*, *SMAD3*, *OTX2*, *BCL2L1* mRNAs in D283 Med cells treated for 72 h with sh_SCR or sh_HMGN5. Expression levels were compared to sh_SCR sample as control, set as 1. Data (means ± SEM) are expressed in arbitrary units and are relative to *GAPDH* mRNA levels. *N* = 3, * *p* ≤ 0.05, ** *p* ≤ 0.01 (two-tailed Student’s *t*-test). **J** Western blot analysis of c-PARP1 (left panel) and PARP1 (right panel) protein levels in D283 Med cells treated for 72 h with sh_SCR or sh_HMGN5. *N* = 3, * *p* ≤ 0.05 (two-tailed Student’s *t*-test). **K** Analysis of number of viable D283 Med cells treated for 72 h with sh_SCR or sh_HMGN5. Data (means ± SEM) are expressed as the number of viable cells, counted by an automated cell counter. *N* = 3, * *p* ≤ 0.05 (two-tailed Student’s *t*-test).

Initially, we hypothesized a straightforward interaction between *lncMB3* and target mRNAs downregulated upon its KD. Preliminary analyses using the IntaRNA algorithm (http://rna.informatik.uni-freiburg.de/), which maps RNA-RNA binding regions, yielded low interaction propensity (Dataset 4). However, to experimentally explore possible indirect associations, we conducted RNA pull-down assays as in [28], with 2 independent pools of specific biotinylated antisense probes (Fig. 4A). qRT-PCR analysis of *lncMB3*-enriched pull-down fractions with EVEN and ODD probe sets compared to control LacZ-precipitated fractions (Dataset 5) showed no significant candidate enrichment (Dataset 6), excluding any RNA interaction between *lncMB3* and TGF-β or downstream pathway transcripts.

We then considered the competing endogenous RNA network, a regulatory mechanism in which long transcripts modulate microRNA activity by acting as molecular sponges. Through this mechanism, *lncMB3* may sequester microRNAs repressing its targets and indirectly control their expression. Preliminary interaction predictions between *lncMB3* and the core RNA-induced silencing complex component Argonaute 2 (AGO2) performed through the *cat*RAPID algorithm (http://s.tartaglialab.com/page/catrapid_group) (Dataset 7) were experimentally validated through a CLIP assay (Fig. S5A). Based on these results, we employed Targetscan (https://www.targetscan.org/vert_80/) to identify microRNAs predicted to bind both *lncMB3* and target mRNAs (Dataset 8). Of 6 candidates (*hsa-miR-135b-5p*, *hsa-miR-216a-3p*, *hsa-miR-3681-3p*, *hsa-miR-613*, *hsa-miR-1197*, *hsa-miR-196b-5p*), the top 3 were tested by qRT-PCR on *lncMB3* pull-down fractions, showing no significant enrichment and ruling out the hypothesis of competition for microRNA association (Dataset 9).

Finally, *lncMB3* may regulate its downstream gene network by binding and modulating other mRNAs, that are in turn involved in the control of target gene expression [29]. To explore this hypothesis, we identified *lncMB3* interactome by cross-referencing RNA-Seq data from independent *lncMB3* pull-down replicates (Fig. 4B and Dataset 10), applying a stringent analysis to pinpoint key binding partners. We found two mRNAs (*HMGN5*, *EIF5B*) and one lncRNA (*ANKDDA1*) associated with *lncMB3* (Figs. 4B and S6A). Notably, the mRNA for the nuclear factor HMGN5 (High Mobility Group Nucleosome Binding Domain 5) had the highest FC and the most significant FDR. Given the broad epigenetic role of HMGN5 in transcriptional regulation [30] and its interplay with *lncMB3*, it can be highlighted as a candidate for further analysis.

### *LncMB3* interacts with *HMGN5* mRNA

The chromatin factor HMGN5 regulates gene expression by influencing nucleosome-DNA interactions [30, 31]. *HMGN5* is ubiquitously expressed (Fig. S6B), with low expression in cerebellum, among brain tissues. RNA-Seq from the primary MB dataset GSE164677 showed a trend of *HMGN5* upregulation in MB (Fig. 4C).

Co-enrichment of *HMGN5* mRNA with *lncMB3* was validated in additional pull-down assays (Fig. 4D). Upon nucleus/cytoplasm subcellular fractionation of D283 Med cell extracts, we also performed cytoplasmic native RNA pull-down assays and highlighted that *lncMB3*/*HMGN5* interaction occurs in this compartment (Fig. S7A). IntaRNA predicted an interaction site (energy score: −30.1 kcal/mol) spanning ~120 nucleotides between *lncMB3* 5’ region (nt 189–307) and *HMGN5* mRNA coding sequence (nt 933–1059) (Fig. 4E). This prediction was experimentally verified using 4′-aminomethyl-4,5′,8-trimethylpsoralen (AMT)-crosslinked RNA pull-down assays [28], which detect direct RNA-RNA pairings in vivo (Fig. 4F). Finally, digital PCR-based absolute quantifications showed stoichiometrically equivalent expression levels for *lncMB3* and *HMGN5* (Fig. S6C).

To assess the functional consequences of this interaction, we investigated *lncMB3* influence on *HMGN5* expression. *HMGN5* mRNA levels remained unchanged following *lncMB3* KD, as per RNA-Seq data (Dataset 1) and qRT-PCR (Fig. S8A). This is further confirmed in vivo by the lack of correlation between *lncMB3* and *HMGN5*, according to the dataset GSE164677 (Spearman Coefficient 0.29). However, HMGN5 protein levels dropped by ∼80% upon *lncMB3* KD (Fig. 4G), consistently with HMGN5 recognised oncogenic activity in various cancers [32, 33]. Restoring *lncMB3* levels through transient exogenous expression of Δ16 construct after one pulse of KD (Fig. S8B), showed a 20% recovery of HMGN5 protein levels, indicating a specific and quantitative regulation of HMGN5 factor by *lncMB3*.

The impact of *lncMB3* on HMGN5 protein and their RNA-RNA interaction indicates that the lncRNA acts as a translational activator. This was confirmed using a Ribo-Tag strategy [34] with transient FLAG-tagged 60S ribosomal protein L22 (RPL22) expression in D283 Med cells (Fig. S8C). Western blot analysis of FLAG-RPL22-precipitated fractions showed comparable protein levels in gap_SCR *vs* gap_*lncMB3* extracts (Fig. S8D). At variance, qRT-PCR analysis revealed a marked decrease in *HMGN5* mRNA association with ribosomes in the absence of *lncMB3*, compared to control (*GAPDH*, Fig. 4H). These results indicate that *lncMB3* is required for *HMGN5* mRNA ribosomal association.

### HMGN5 regulates TGF-β pathway and apoptosis in G3 MB cells

Given the documented HMGN5 protein nuclear activity, we re-evaluated genes altered upon *lncMB3* KD to assess if their expression changes were mainly transcriptional. qRT-PCR analysis was performed with exon-exon *vs* exon-intron junction primers to differentiate mature mRNAs from primary transcripts. As shown in Fig. S8E, *lncMB3* silencing downregulated *ACVR1B*, *INHBE*, *SMAD3*, *OTX2*, *NRL* and *CRX* pre-mRNAs, highlighting a nuclear control over several genes regulated by *lncMB3* (compare Figs. 1B, 1C, 2B and 3A).

HMGN5 role in suppressing apoptosis had not previously been assessed in MB. To functionally connect *lncMB3* and HMGN5 activities, we aimed to mimic the molecular effects of *lncMB3* KD by silencing *HMGN5*. Using VectorBuilder (https://en.vectorbuilder.com/), we designed a short interfering RNA targeting *HMGN5*. Transient transfections in D283 Med cells resulted in a ~ 30% downregulation of *HMGN5* mRNA (Fig. 4I) and a ~ 60% reduction in protein levels (Fig. S8F). This led to a 20–50% RNA decrease for *INHBE*, *SMAD3*, *OTX2* and *BCL2L1* (Fig. 4I), targets also downregulated upon *lncMB3* KD (see Figs. 1B, 1C, 2B and 3A), indicating that *HMGN5* KD appreciably phenocopies *lncMB3* molecular effects (Fig. S8G). Regarding cell death, *HMGN5* KD caused a threefold increase in c-PARP1 protein (Fig. 4J, left panel) and a 30% decrease in full-length PARP1 (Fig. 4J, right panel). HMGN5 level reduction also mirrored a viable D283 Med cell number decrease, with a drop of ~ 30% (Fig. 4K), similar to the impact of *lncMB3* targeting. These findings demonstrate that *lncMB3* and HMGN5 participate into the same molecular and cellular phenotypes. Dependent on interactions with *lncMB3*, *HMGN5* mRNA decreased translation contributes to the nuclear regulation of TGF-β pathway and downstream gene module in G3 MB cells.

### *LncMB3* inhibition synergizes with DDP treatment in G3 MB cells

DDP is a first-line anti-cancer agent and a cornerstone of MB treatment [35]. Combining therapies can reduce DDP side effects and fight both intrinsic and acquired resistance. Given the shared influence of *lncMB3* and DDP on apoptosis-related pathways [36], we asked whether *lncMB3* KD could amplify DDP cytotoxicity in G3 MB cells.

**Fig. 5:**
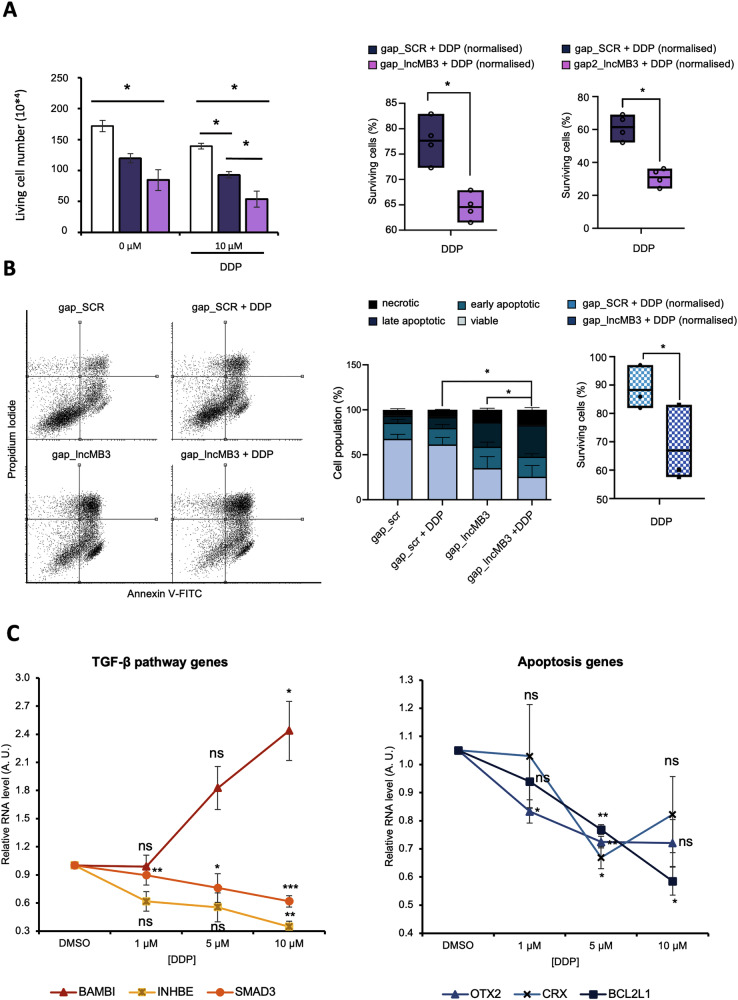
Analysis of *lncMB3* KD/DDP treatment on D283 Med cell viability and apoptosis. **A** Left Panel: dose-response analysis of the number of viable D283 Med cells upon DDP treatment. Cells were treated with the vehicle (DMSO), 10 μM/mL DDP, untreated (NT) or treated with gap_SCR or gap_*lncMB3* (GapmeR #1). *N* = 4, * *p* ≤ 0.05, (two-tailed Student’s *t*-test). Right Panel: Percentage of viable D283 Med cells following DDP administration and *lncMB3* GapmeR transfection (GapmeR #1 or GapmeR #2). Double-treated samples (gap_SCR + DDP and gap_*lncMB3* + DDP) were normalised on the viable cell number of the corresponding transfected-only sample (gap_SCR and gap_*lncMB3*, respectively). DDP concentration used was 10 μM. *N* = 4, * *p* ≤ 0.05 (two-tailed Student’s *t*-test). **B** Left panel: representative flow cytometry analysis of PI− and Annexin V-stained D283 Med cells following DDP administration [1 μM] and GapmeR #1 transfection 24 h after *lncMB3* KD, with or without DDP treatment. Middle panel: quantification of viable (Annexin V−/PI−), early apoptotic (Annexin V+/PI−), late apoptotic (Annexin V+/PI+) and necrotic (Annexin V−/PI+) fractions. *N* = 3, * *p* ≤ 0.05 (comparison between early + late apoptotic cells). Right panel: percentage of viable D283 Med cells. Data normalisations as in (**A**). *N* = 3, * *p* ≤ 0.05 (two-way ANOVA). **C** qRT-PCR analysis of TGF-β pathway genes (left panel) and downstream pathway genes (right panel) in D283 Med cells treated for 24 h with DDP at different doses (reported on the x-axis). Control cells were treated with vehicle (DMSO) and set as 1. Data (means ± SEM) are expressed in arbitrary units and are relative to *GAPDH* mRNA levels. *N* = 3, * *p* ≤ 0.05, ** *p* ≤ 0.01, *** *p* ≤ 0.001 (two-tailed Student’s *t*-test).

We first performed dose-response assays for each drug treatment (DDP or GapmeR #1), to evaluate their effect on the reduction of D283 Med cell viability (Fig. S9A). Based on these results, we then combine 10 μM DDP administration, with 100 nM GapmeR #1 and assessed the number of surviving cells (Fig. 5A, left panel). Normalising double-treated *vs* drug-untreated samples, cell counts revealed lower relative survival rate in gap_*lncMB3*-treated cells compared to scramble-transfected, indicating a synergistic effect between treatments (Fig. 5A, right panels). This finding was confirmed using an equivalent setup with 100 nM GapmeR # 2 (Fig. 5A, right panels). To further validate this effect, we conducted an apoptosis assay with Annexin V staining (Fig. 5B, left panel). Quantification of non-viable *vs* viable cells showed an increase of late apoptotic cell number compared to other populations upon combined treatments (Fig. 5B, middle panel), reinforcing the synergistic effect (Fig. 5B, right panel).

To examine whether synergy relied on a crosstalk between treatments, we tested DDP administration consequences on *lncMB3* target genes. A partial deregulation of both the TGF-β (Fig. 5C, left panel) and apoptotic pathway genes (Fig. 5C, right panel) was highlighted, similar to *lncMB3* KD. *LncMB3* expression remained unaffected by chemotherapy (Fig. S9B), suggesting the two treatments as independent triggers with overlapping outcomes.

The synergistic enhancement of anticancer effects was not indiscriminate. When GapmeR #1 was combined with Vincristine, another MB therapeutic drug [37] whose autonomous effects on D283 Med cells are reported in Fig. S9A, only additive, not synergistic effects were measured on viable cell counts (Fig. S9C) and apoptosis (Fig. S9D). To formally quantify all these drug interactions, we finally applied the Chou-Talalay method [38], calculating the Combinatorial Index (CI) between administration of GapmeR #1 and each of the two drugs. A synergistic effect (CI < 1) was confirmed with DDP, while an additive effect was validated with Vincristine (CI≈1) (Fig. S9E).

These data suggest that, in vitro, *lncMB3* targeting potentiates DDP treatment effectiveness in reducing G3 MB cell proliferation and inducing apoptosis.

### Production of the ferritin variant HFt-HIS-PASE

Targeting *lncMB3* affects the TGF-β pathway, enhances apoptosis, and boosts chemotherapeutic efficiency in cancer cell lines. This led us to explore the use of human ferritin nanoparticles (NPs) to formulate innovative RNA/protein complexes aimed at *lncMB3*-directed GapmeR delivery. Human ferritin H-type (HFt) is a promising NP for drug delivery, particularly chemotherapeutics, due to its internalisation by the transferrin receptor 1 (CD71), frequently overexpressed in tumours [13, 39].

We designed and synthesized HFt-HIS-PASE, a recombinant ferritin variant derived from a prior HFt version, HFt-MP-PASE [40]. HFt-HIS-PASE contains a tumour-selective sequence (MP), responsive to tumour protease MMP-2 and -9 activities, and a shielding polypeptide (PASE) that enhances stability, masks the surface, and increases target specificity. Additionally, HFt-HIS-PASE features a five-histidine motif known to aid nucleic acid endosomal escape [41] (Fig. S10A).

**Fig. 6:**
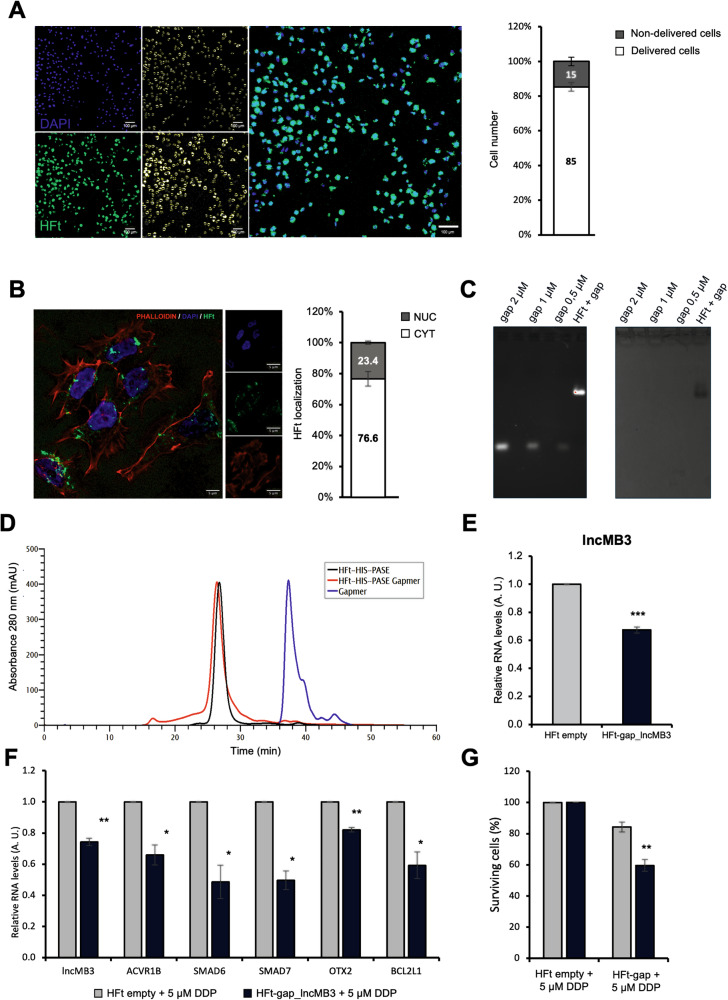
Synthesis, analysis and application of HFt-HIS-PASE/GapmeR complexes. **A** Left panel: representative image of colocalisation analysis between fluorescein-HFt-HIS-PASE and DAPI in D283 Med cells, 12 h after administration (magnification 20×). Fluorescein-HFt-HIS PASE (green) and DAPI (blue, nuclear staining) are on the left, object counts after binarisation are in the middle and the merge of the two channels is on the right. Right panel: percentage of colocalisation between fluorescein-HFt-HIS-PASE and DAPI in D283 Med cells. Data (mean ± SEM) were obtained from 8 different fields. **B** Left panel: representative immunofluorescence of fluorescein-HFt-HIS PASE delivered for 24 h (green), DAPI (blue, nuclear staining) and phalloidin (red, cytoplasmic staining) (magnification 60×). Right panel: percentage of nucleus/cytoplasm subcellular localisation of HFt-HIS PASE in D283 Med cells. Data (mean + SEM) were obtained from 5 different fields. **C** Band migration profiles on agarose gel electrophoresis. Gel was double-stained with SYBR Gold for GapmeR visualisation (left panel) and Coomassie Blue for ferritin visualisation (right panel). *Lane 1*: GapmeR standard 2 µM; *Lane 3*: GapmeR standard 1 µM; *Lane 5*: GapmeR standard 0.5 µM; *Lane 6*: HFt-HIS-PASE-GapmeR complex. **D** Size Exclusion Chromatography profile analysis of HFt-HIS-PASE (in black), HFt-HIS-PASE-GapmeR #1 complexes (in red) and GapmeR #1 alone (in blue). **E** qRT-PCR analysis of *lncMB3* levels in D283 Med cells upon treatment with HFt-HIS-PASE-GapmeR #1 complexes, 48 h after delivery, compared with empty HFt-HIS-PASE, set as 1. Data (means ± SEM) are expressed in arbitrary units and are relative to *GAPDH* mRNA levels. *N* = 3, *** *p* ≤ 0.001 (two-tailed Student’s *t*-test). **F** qRT-PCR analysis of TGF-β pathway genes in D283 Med cells treated for 48 h with HFt-HIS-PASE-GapmeR #1 + 5 µM DDP. Expression levels were compared to HFt-HIS-PASE-empty + 5 µM DDP, set as 1. Data (means ± SEM) are expressed in arbitrary units and are relative to *GAPDH* mRNA levels. *N* = 3, * *p* ≤ 0.05, ** *p* ≤ 0.01 (two-tailed Student’s *t*-test). **G** Number of viable D283 Med cells treated for 48 h with HFt-HIS-PASE-GapmeR #1 + 5 µM DDP. HFt-HIS-PASE empty + 5 µM DDP was used as control. Data (means ± SEM) are expressed as the percentage of viable cells, counted by an automated cell counter. *N* = 4, ** *p* ≤ 0.01 (two-tailed Student’s *t*-test). Where necessary in the figure HFt-HIS-PASE is referred as HFt.

Preliminary qRT-PCR assessments of *MMP-2*, *MMP-9* and *CD71* mRNA abundance in HD-MB03 and D283 Med cells (Fig. S10B) led to selecting the latter for further tests. We then evaluated the targeting capacity of a Fluorescein-5-Maleimide-labeled HFt-HIS-PASE nanovector (HFt-HIS-PASE-fluo). About 85% of D283 Med cells exposed to HFt-HIS-PASE-fluo (1 mg/mL) showed fluorescence signal 12 h post-administration, indicating the affinity of HFt-HIS-PASE for MB cells (Fig. 6A). Immunofluorescence assays performed 24 h later, using nuclear (DAPI) and cytoplasmic (Phalloidin) staining and confocal microscopy analysis, demonstrated intracellular uptake of HFt, with a nuclear-cytoplasmic distribution (Fig. 6B).

### Analysis of HFt-HIS-PASE-GapmeR complexes and *lncMB3* targeting

To evaluate *lncMB3* targeting using HFt-ASO complexes, we generated and characterised HFt-HIS-PASE-GapmeR nucleoprotein particles. The *lncMB3* GapmeR #1 was encapsulated in the internal cavity of HFt-HIS-PASE via pH-dependent HFt dissociation/reassociation. Successful GapmeR loading was confirmed by electrophoresis of purified HFt-HIS-PASE-GapmeR complexes. Distinct migration patterns for the complex compared to free GapmeR were observed. DNA-specific staining (Fig. 6C, left panel) displayed significant delay for nucleic acid when associated with HFt-HIS-PASE (lane HFt + gap), while protein-specific staining confirmed a co-migration profile between HFt-HIS-PASE-GapmeR complex and ferritin (Fig. 6C, right panel).

Regarding stability, no nucleic acid degradation was observed after 1 h and overnight incubations of HFt-HIS-PASE-GapmeR with DNA/RNA nuclease at 37 °C (Fig. S10C). No degradation was noted after 3 months of storage at 4 °C, indicating GapmeR encapsulation within the protein cavity, shielded from external exposure.

To evaluate GapmeR binding efficiency to HFt NPs, the relative intensities of agarose bands corresponding to DNA or protein were quantified. Comparisons were made with free GapmeR and HFt-HIS-PASE molecules at standard concentrations. The final GapmeR/HFt-HIS-PASE molecular ratio was 0.5:1. Purity and hydrodynamic volume of HFt-HIS-PASE-GapmeR were determined by size-exclusion chromatography, showing similar elution volume and size to non-encapsulated HFt-HIS-PASE protein, with no free GapmeR detected, indicating co-elution with ferritin (Fig. 6D).

The biological activity of the complex was tested in D283 Med cells treated with a single dose of HFt-HIS-PASE alone or GapmeR-complexed (200 nM). Steady-state *lncMB3* levels, 48 h post-treatment, showed a 30% reduction with encapsulated GapmeR #1 compared to mock-treated cells (Fig. 6E). Similar outcomes were obtained with HFt-HIS-PASE-GapmeR stored at 4 °C for 3 months, replicating the effects of a standard, single-pulse GapmeR #1 transfection at 100 nM for 48 h (Fig. S10D). No modulation of TGF-β pathway gene expression, cell proliferation reduction, or apoptosis increase was registered under these conditions, probably due to insufficient *lncMB3* downregulation. However, to further investigate HFt-HIS-PASE-mediated cellular effects of *lncMB3* targeting, we leveraged our findings on the synergy between *lncMB3* KD and DDP administration. D283 Med cells were treated with 5 μM DDP alone or in combination to HFt-HIS-PASE-GapmeR. Combined treatments led to a 20–50% reduction in TGF-β pathway and downstream genes (Fig. 6F), as revealed by qRT-PCR analysis. A decrease in cancer cell survival was also observed, compared to DDP treatment alone (Fig. 6G), consistent with earlier data (Fig. 5A), indicating that HFt-based NPs are effective for *lncMB3* targeting and sensitising G3 MB cells to anticancer drugs.

## Discussion

Integrated multi-omics has combined molecular genetic with histological analyses within a framework to illuminate MB inherent heterogeneity and complexity [1]. Despite unique genomic, biochemical and clinical traits in each MB subgroup [42], therapies still rely on general interventions—surgical resection, chemotherapy and radiotherapy—with 5-year survival rates of 60%–80% [43]. This underscores the need for targeted therapies and deeper explorations of dysfunctional pathways, particularly in high-risk and poorly characterised subgroups, like G3 and G4. G3 MB, representing 25% of cases, exhibits the highest recurrence and metastasis rate, correlating with worse outcomes [44]. The discovery of *MYC* amplification/overexpression as a major G3 MB pathogenic factor [45, 46] aligns with estimates of aberrant MYC activity in 70% of human cancers [46]. Yet, targeting MYC has long been challenging due to its disordered structure, leading to low-potency therapeutic candidates, with suboptimal pharmacological properties and considerable off-target effects [47]. Furthermore, MYC plays a critical role in physiological processes including differentiation, proliferation, and cell cycle regulation, which are central to development and post-natal life. This makes it essential to develop strategies for MYC inhibition, particularly in paediatric cancers. Several pre-clinical studies and clinical trials are investigating direct MYC inhibitors [47, 48], like OMOMYC, a dominant-negative showing promise in solid cancers [10, 48], that we used to map the MYC transcriptome in G3 MB cells [10]. Indirect strategies targeting MYC cofactors or regulators are valuable alternatives and, along this direction, understanding MYC-dysregulated gene pathways, including non-coding RNA networks, may diversify and improve opportunities for MYC-targeted therapies [49].

Over the past two decades, the rise of non-coding RNAs has reshaped cancer biology [50]. LncRNAs have gained attention as cancer hallmark modulators for their tissue- and cell-specific expression and their structural flexibility, making them attractive biomarkers and molecular targets. However, their potential as biological master regulators require detailed functional characterisation.

To date, only few lncRNAs have been mechanistically described in G3 MB. This study clarifies the role of MYC-regulated *lncMB3* in G3 medulloblastomagenesis, establishing a foundation for its potential application. *LncMB3* is upregulated in G3 MB cell lines and primary tumours, playing a pivotal role in cell death evasion in vitro. Through transcriptomic, molecular and cellular assays, we show that *lncMB3* regulates the TGF-β cascade, crucial for G3 MB pathology, impacting cancer cell proliferation and survival [11]. Notably, we identified *lncMB3* target gene cluster in this pathway, whose misregulation elevates the retinal TF OTX2, an oncogenic driver in G3 MB that boosts cell proliferation, suppresses apoptosis, and contributes to tumour aggressiveness [51]. OTX2 and other photoreceptor TFs, including NRL and CRX, drives anti-apoptotic factors like BCL2L1 [23], linking this gene program to sustained cancer cell viability. *LncMB3* activity orchestrates key pathways, genes, and processes in G3 MB, forming a nexus among *MYC* driver gene, TGF-β signalling, photoreceptor gene network, and apoptosis regulation. This interconnection offers an integrated framework for understanding the molecular underpinnings of this tumour subgroup.

While our coding transcriptome analysis provided insights into *lncMB3* function, its interactome revealed its mode of action. *LncMB3* directly interacts with *HMGN5* mRNA, encoding an epigenetic regulator within the HMGN nucleosome-binding and nuclear architecture family, involved in extensive chromatin decondensation and transcriptional regulation [32, 33]. HMGN5, implicated in embryonic gene regulation [52] and exhibiting oncogenic properties in several cancers [32, 33], shows a tendency toward upregulation in MB. Our findings suggest that functional RNA-RNA interactions between coding and non-coding molecules—an emerging theme in cancer regulation—upregulate HMGN5 protein levels, explaining why *HMGN5* RNA interference phenocopies *lncMB3* targeting effects. Given that several *lncMB3* targets are dysregulated in the nucleus, where HMGN5 protein operates, this mechanism adequately accounts for the observed molecular and cellular phenotypes, though additional regulative cascades or secondary effects could exist, consistent with multifunctional scaffolding properties and diverse subcellular localisation of lncRNAs. Owing to its molecular features—ranging from dual nucleocytoplasmic distribution to chromatin-independent regulatory activity—*lncMB3* can be regarded as a trans-acting lncRNA. Along this line, *lncMB3*/*HMGN5* co-expression confined to pathological cerebellum suggests their interaction as a platform for developing novel inhibitors competing out disease-specific complexes.

Due to G3 MB aggressive nature and treatment resistance, integrated therapies promise solutions. ASOs have gained prominence in RNA-based treatments for their design flexibility and pharmacological properties [50]. Our analysis of *lncMB3*-targeting LNA GapmeRs combined with standard MB chemotherapy demonstrated synergistic interplay, enhancing drug cytotoxicity. This effect likely stems from both treatments acting on the intrinsic apoptotic pathway [36] and the TGF-β cascade (this study). These findings suggest modulating the *lncMB3* molecular network to amplify anticancer agent efficacy, to support RNA-based combinatorial strategies to reduce tumour resistance and to improve G3 MB treatment outcomes. However, a major challenge in RNA therapies is ensuring efficient effector delivery to intended sites. We explored application of HFt-based nanocarriers for deploying LNA GapmeRs targeting *lncMB3*. HFt properties, from high biocompatibility and stability to low toxicity and cost-effectiveness, make it a powerful drug delivery system [13]. Moreover, HFt capacity to cross the blood-brain barrier [13] positions it as a promising tool for brain tumour therapies. Its symmetrical self-assembly, small and uniform size, and versatile surface functionalisation are ideal for bioactive compound delivery, including small nucleic acids. While siRNAs [53] and microRNAs [54] have already been delivered by HFt, this study is the first, to our knowledge, to employ HFt-encapsulated ASOs for gene silencing. Combinatorial administration of *lncMB3*-targeting NPs and DDP replicated the synergistic effects observed with ASO transfection, impacting the TGF-β pathway and reducing cell survival. These results indicate that HFt-based strategies have potential for targeting oncogenic pathways at the RNA level via biocompatible delivery of tumour-specific antisense oligomers.

**Fig. 7:**
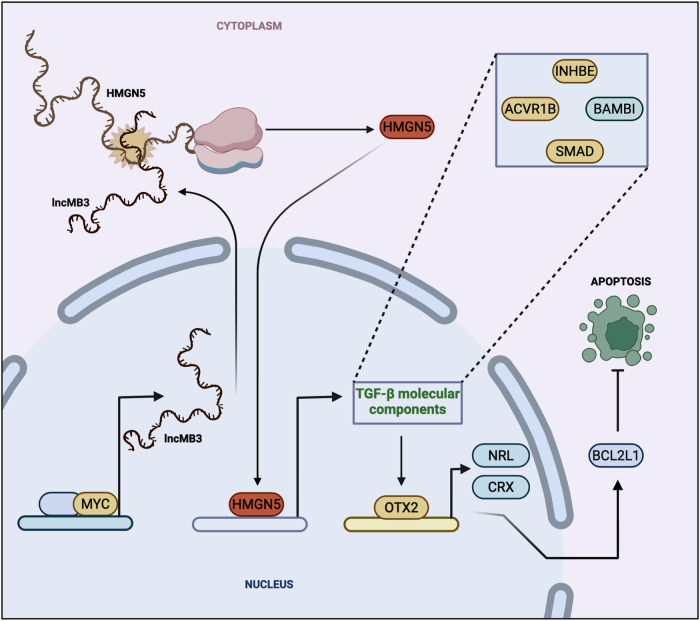
Regulatory circuit. Schematic representation of the MYC-dependent *lncMB3* mechanism of action. Image made with Biorender (https://biorender.com).

In conclusion, this study highlights the critical role of *lncMB3* in G3 MB apoptosis regulation through TGF-β pathway modulation and interaction with underexplored genes, such as *HMGN5*. *LncMB3* stands out as a key regulatory node linking *MYC* amplification to enhanced tumour cell survival via cell death inhibition (Fig. 7). Future efforts will focus on deeper exploration of *lncMB3* interactions and regulatory mechanisms-of-action, as well as validating its therapeutic potential in preclinical G3 MB models using the HFt nanocarriers.

## Materials and Methods

All cell lines were obtained from ATCC and cultured as previously described [10]. Evaluation of viable cell number was assessed after cell manipulations by CytoSMART Cell Counter. Gene silencing or overexpression were realised transfecting antisense LNA GapmeRs or plasmid constructs by Lipofectamine 2000 as per manufacturer’s instructions. Expression of specific RNAs was performed by standard qRT-PCR of digital PCR. TruSeq Stranded mRNA Library Prep Kit was used to obtain sequencing libraries from polyA+ RNA. RNA-Seq analyses were performed by conventional R package DESeq2 (Bioconductor), Gene Ontology and Gene Set Enrichment Analysis. Protein expression was assessed by conventional immunoblotting, detected by ChemiDoc XRS+ Molecular Imager and quantified through the Image Lab Software. Chemotherapeutics (cisplatin, Vincristine) were administered at different concentrations according to the specific experimental plan, eventually in combination with GapmeR transfection. Apoptosis was analysed by determination of Annexin V-FITC/PI staining through flow cytometry. Data were analysed using the Flowing software. Alternatively, cell death markers were analysed by immunoblot assay. For Crosslinked Immunoprecipitation assay, cytoplasmic extracts were incubated with AGO2 antibody or IgG and coupled to Protein G Dynabeads resin. Immunoprecipitated proteins were collected in RIPA buffer and analysed. Immunoprecipitated RNA was treated by Proteinase K before qRT-PCR. RNA Pull Down experiments were performed as described in [28] from endogenous cell extracts using separate sets of antisense biotinylated probes binding to streptavidin Magnasphere paramagnetic beads. Pull Down-Seq analysis were performed through edgeR to quantify significant genes, using a generalised linear model. RNA-RNA interaction prediction was computed using IntaRNA 3.3.2, whereas TargetScan Human database was exploited to obtain candidates microRNAs. Ribotagging and RNA Immunoprecipitation was performed as described in [34], exploiting exogenous expression of a FLAG-tagged version of the RPL22 protein for enriching the ribosomal fraction from cell lysates. HFt-HIS-PASE was obtained as a recombinant protein considering codon optimisation for high expression levels in *E. coli*. BL21 (DE3) strain. Size-exclusion chromatography experiments were performed using a Superose 6 gel-filtration column. The HFt-HIS-PASE protein was incubated with Fluorescein-5-Maleimide for fluorescent labelling. Electrophoresis on agarose gel of the purified HFt-GapmeR complexes was used to demonstrate GapmeR loading. For nucleocytoplasmic staining, fluo-HFt targeted cells were fixed in paraformaldehyde, permeabilised and blocked with Triton ×-100/BSA/PBS. Fixed cells were incubated with Alexa Fluor™ 555 Phalloidin and DAPI solution. For confocal microscopy experiments, samples were imaged using an Olympus iX83 FluoView1200 laser scanning confocal microscope. The Fiji “Analyse Particles” tool was employed for cell counts, whereas signal co-localisation was analysed using the “JACoP” Fiji plugin. HFt-ASO complexes were directly administered to cells, according to the specific experimental plan. Oligonucleotide, probe and GapmeR sequences are listed in Dataset 5. For detailed technical descriptions, see “Supplementary Materials and Methods”.

## Supplementary information

Supplementary Figures and legends

Supplementary Materials and Methods

Uncropped wb

dataset 1

dataset 2

dataset 3

dataset 4

dataset 5

dataset 6

dataset 7

dataset 8

dataset 9

dataset 10

## Data Availability

The RNA-Seq data presented in this study are available in GEO, accession numbers GSE277976 and GSE278666. Uncropped western blots are listed in Dataset—Uncropped Western Blots.
