# Supplementary material for: The MYC-dependent lncRNA MB3 inhibits apoptosis in Group 3 Medulloblastoma by regulating the TGF-β pathway via HMGN5
Source: Cell Death Dis. 2025 Nov 6;16(1):800. doi: 10.1038/s41419-025-08097-8 (PMC12592558; doi:10.1038/s41419-025-08097-8)
Supplement: Supplementary file 1 — Supplementary Figures and legends [file 41419_2025_8097_MOESM1_ESM.pdf]

# S1A

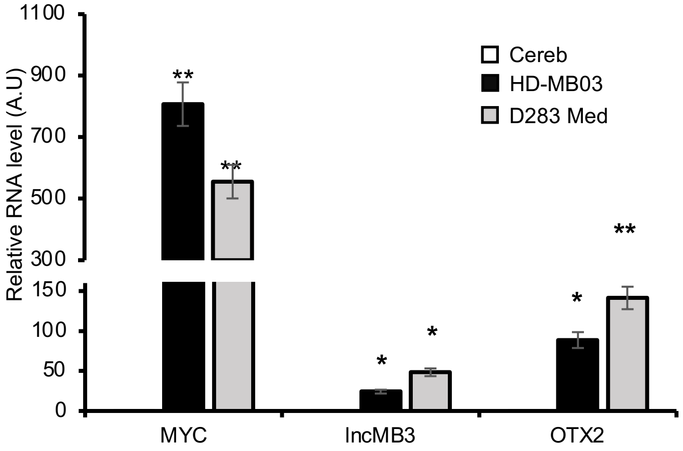


# S1B

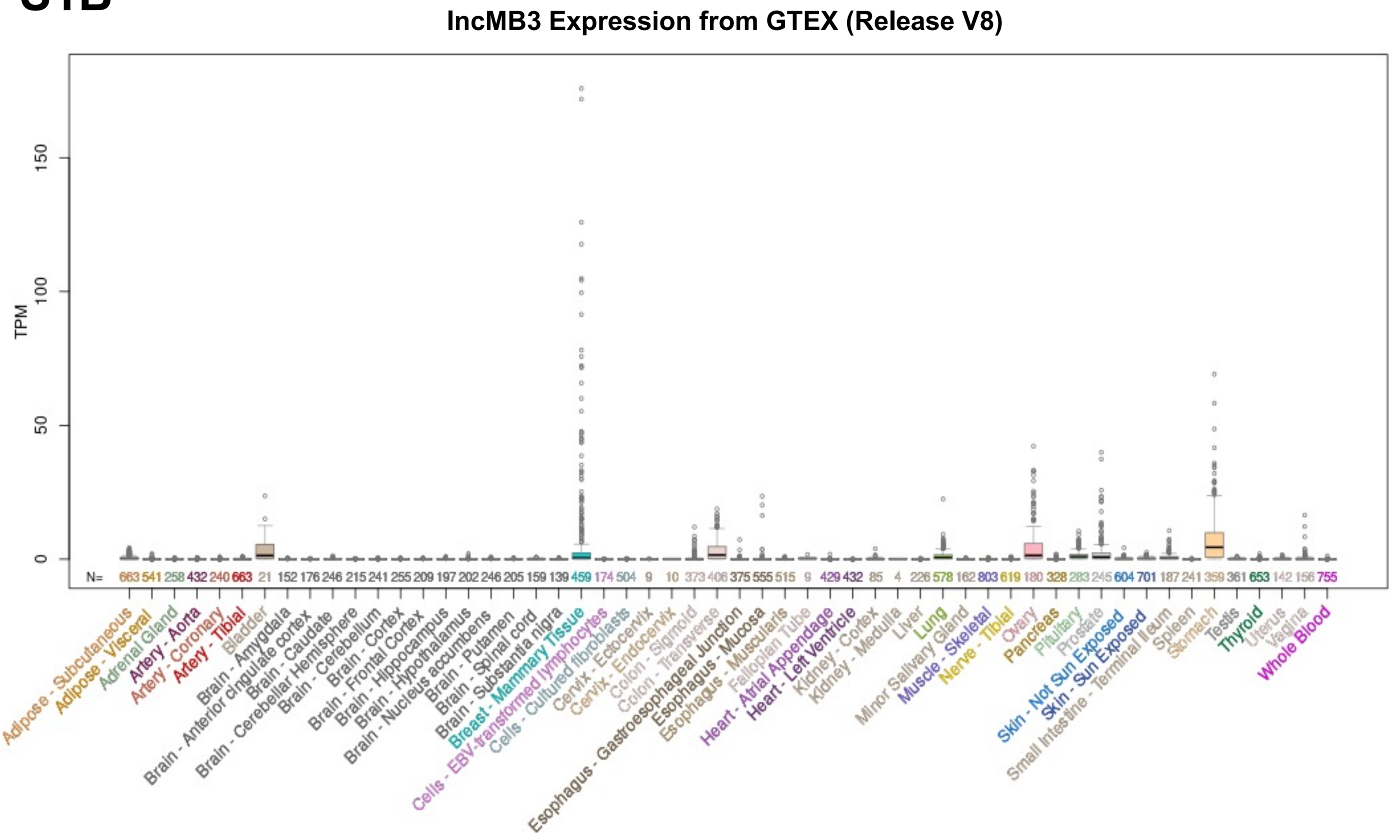


**Figure S1. *LncMB3* expression – Referred to Figure 1 and 2**

**A.** qRT-PCR analysis of *MYC, lncMB3 and OTX2* in a pool of 10 healthy cerebella (Cereb) set as 1, in D283 Med cells or in HD-MB03 cells. Data (means ± SEM) are expressed in arbitrary units (A.U.) and are relative to *GAPDH* mRNA levels. $N = 3$, * $p \leq 0.05$, ** $p \leq 0.01$ (two- tailed Student's *t*-test).

**B.** Expression of *lncMB3* from 54 non-diseased tissues, according to Genotype-Tissue Expression (GTEx) Analysis, Release V8. Levels are shown in Transcripts per Million (TPM). Circles represent outliers. Details at https://gtexportal.org/home/gene/RP11-95I16.6.

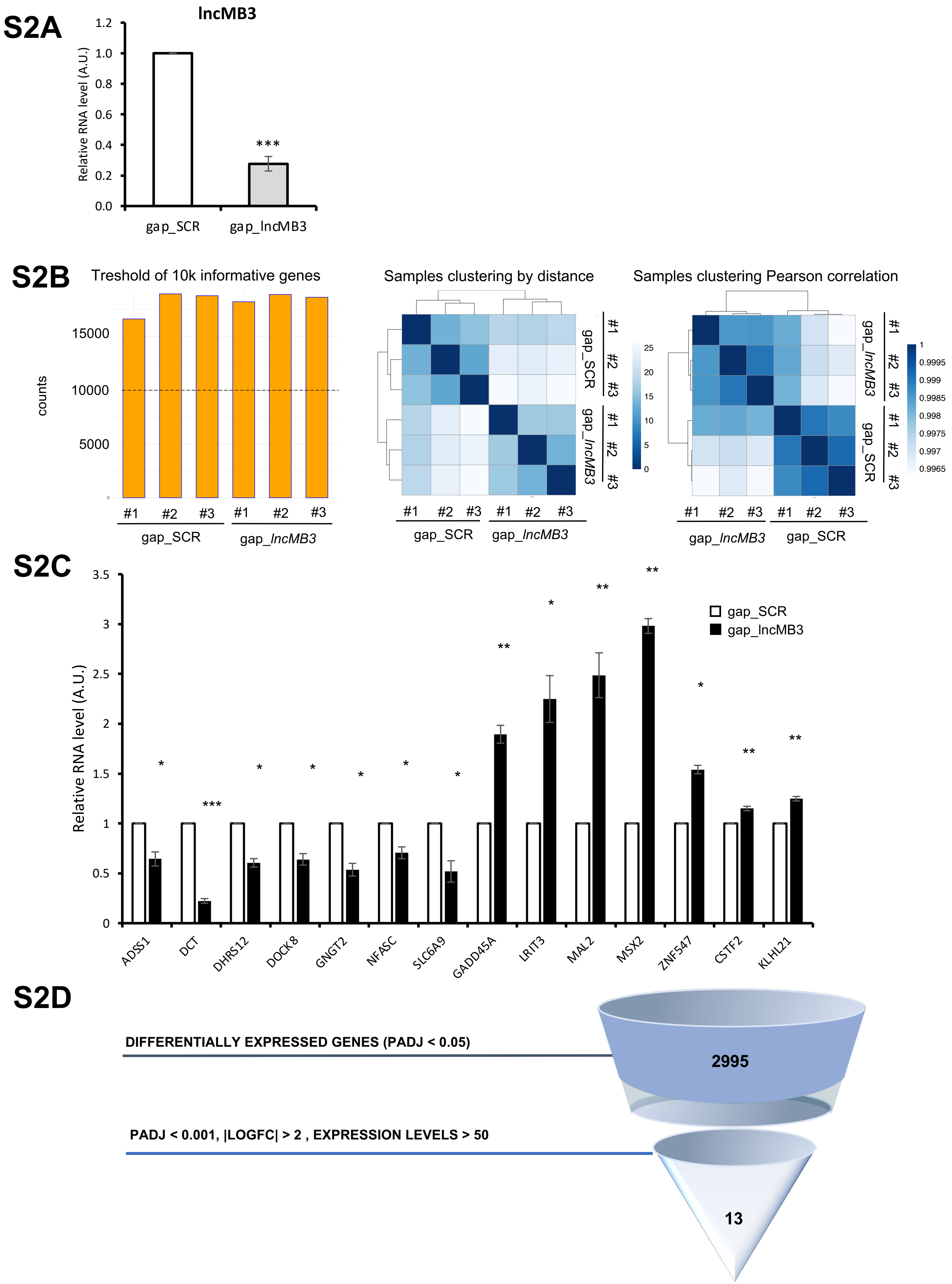
S2A
lncMB3
Relative RNA level (A.U.)
gap_SCR
gap_lncMB3
S2B
Treshold of 10k informative genes
counts
Samples clustering by distance
Samples clustering Pearson correlation
gap_SCR
gap_lncMB3
S2C
Relative RNA level (A.U.)
gap_SCR
gap_lncMB3
ADSS1
DCT
DHRS12
DOCK8
GNGT2
NFASC
SLC6A9
GADD45A
LRIT3
MAL2
MSX2
ZNF547
CSTF2
KLHL21
S2D
DIFFERENTIALLY EXPRESSED GENES (PADJ < 0.05)
2995
PADJ < 0.001, |LOGFC| > 2 , EXPRESSION LEVELS > 50
13

**Figure S2. *LncMB3*-dependent transcriptomic analysis in G3 MB cells – Referred to Figure 1**

**A.** qRT-PCR analysis of *lncMB3* in gap_SCR samples *vs* gap_*lncMB3* (GapmeR #1) samples. *LncMB3* expression levels upon KD was compared to gap_SCR condition, set as 1. Data (means ± SEM) are expressed in arbitrary units and are relative to *GAPDH* mRNA levels. $N = 3$, *** $p \leq 0.001$ (two- tailed Student's *t*-test).

**B.** Left panel: Number of detected genes obtained from RNA-Seq analysis. Right Panels: Euclidean distance heatmap showing the similarity in gene expression within the SCR and KD (GapmeR #1) samples. Pearson correlation heatmap showing the similarity in gene expression within the SCR and KD (GapmeR #1) samples.

**C.** qRT-PCR analysis of RNA-Seq deregulated transcripts with padj spanning from 0.0091 to 0.046 in gap_SCR *vs* gap_*lncMB3* (GapmeR #1) samples. Expression levels upon KD were compared to gap_SCR condition, set as 1. Data (means ± SEM) are expressed in arbitrary units and are relative to *GAPDH* mRNA levels. $N = 3$, * $p \leq 0.05$, ** $p \leq 0.01$, *** $p \leq 0.001$ (two- tailed Student's *t*-test).

**D.** Schematic representation of DEG quantitative selection.

**S3A**

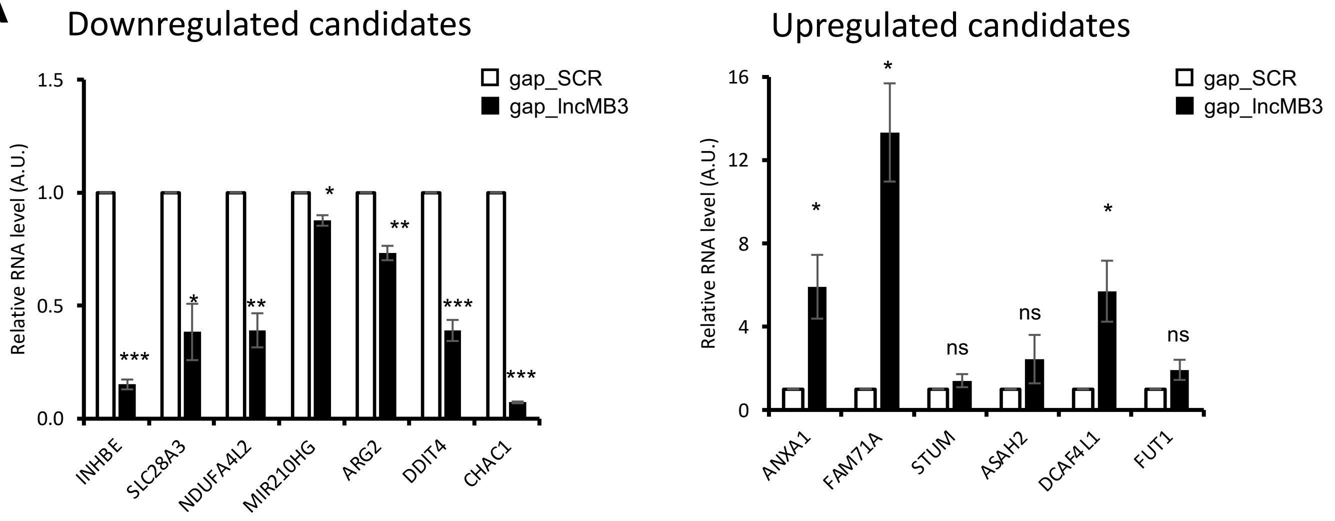
Downregulated candidates
Relative RNA level (A.U.)
1.5
1.0
0.5
0.0
gap_SCR
gap_lncMB3
INHBE
SLC28A3
NDUFA4L2
MIR210HG
ARG2
DDIT4
CHAC1
***
*
**
*
**
***
***
Upregulated candidates
Relative RNA level (A.U.)
16
12
8
4
0
gap_SCR
gap_lncMB3
ANXA1
FAM71A
STUM
ASAH2
DCAF4L1
FUT1
*
*
ns
ns
*
ns

**S3B**

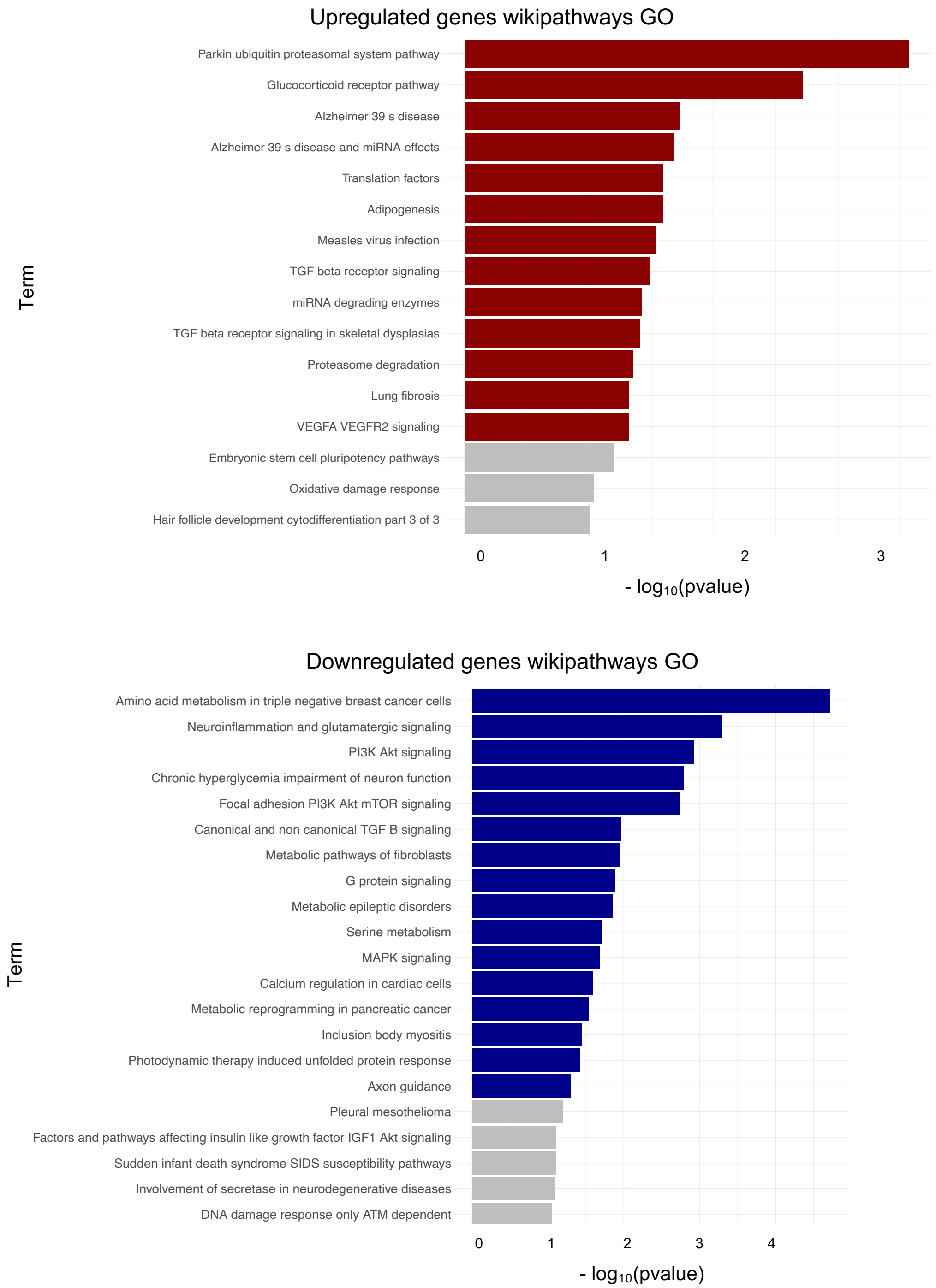
Upregulated genes wikipathways GO
Term
Parkin ubiquitin proteasomal system pathway
Glucocorticoid receptor pathway
Alzheimer 39 s disease
Alzheimer 39 s disease and miRNA effects
Translation factors
Adipogenesis
Measles virus infection
TGF beta receptor signaling
miRNA degrading enzymes
TGF beta receptor signaling in skeletal dysplasias
Proteasome degradation
Lung fibrosis
VEGFA VEGFR2 signaling
Embryonic stem cell pluripotency pathways
Oxidative damage response
Hair follicle development cytodifferentiation part 3 of 3
0
1
2
3
- log10(pvalue)
Downregulated genes wikipathways GO
Term
Amino acid metabolism in triple negative breast cancer cells
Neuroinflammation and glutamatergic signaling
PI3K Akt signaling
Chronic hyperglycemia impairment of neuron function
Focal adhesion PI3K Akt mTOR signaling
Canonical and non canonical TGF B signaling
Metabolic pathways of fibroblasts
G protein signaling
Metabolic epileptic disorders
Serine metabolism
MAPK signaling
Calcium regulation in cardiac cells
Metabolic reprogramming in pancreatic cancer
Inclusion body myositis
Photodynamic therapy induced unfolded protein response
Axon guidance
Pleural mesothelioma
Factors and pathways affecting insulin like growth factor IGF1 Akt signaling
Sudden infant death syndrome SIDS susceptibility pathways
Involvement of secretase in neurodegenerative diseases
DNA damage response only ATM dependent
0
1
2
3
4
- log10(pvalue)

## S3C

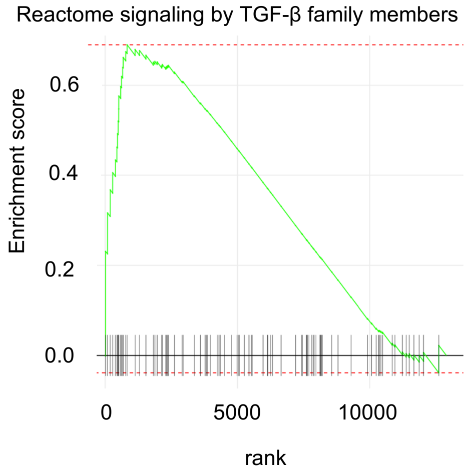
Reactome signaling by TGF-β family members
Enrichment score
0.6
0.4
0.2
0.0
0
5000
10000
rank

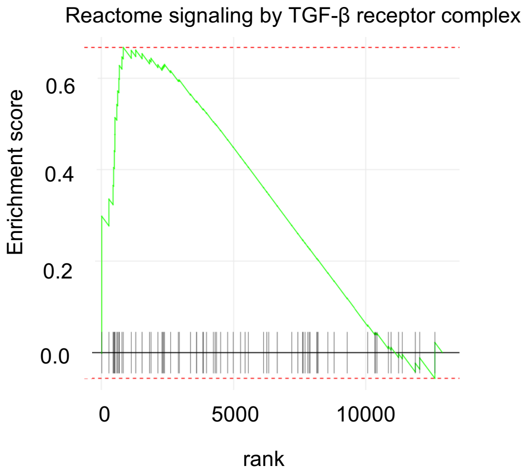
Reactome signaling by TGF-β receptor complex
Enrichment score
0.6
0.4
0.2
0.0
0
5000
10000
rank

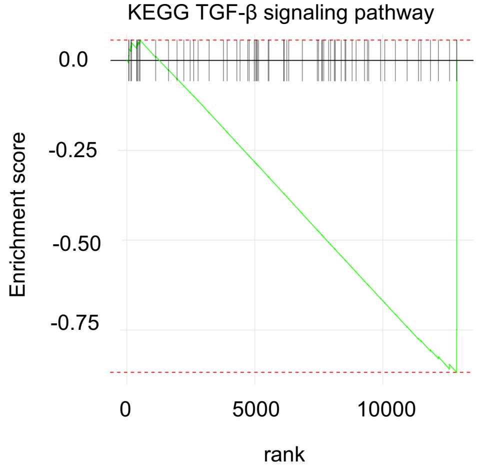
KEGG TGF-β signaling pathway
Enrichment score
0.0
-0.25
-0.50
-0.75
0
5000
10000
rank

## S3D

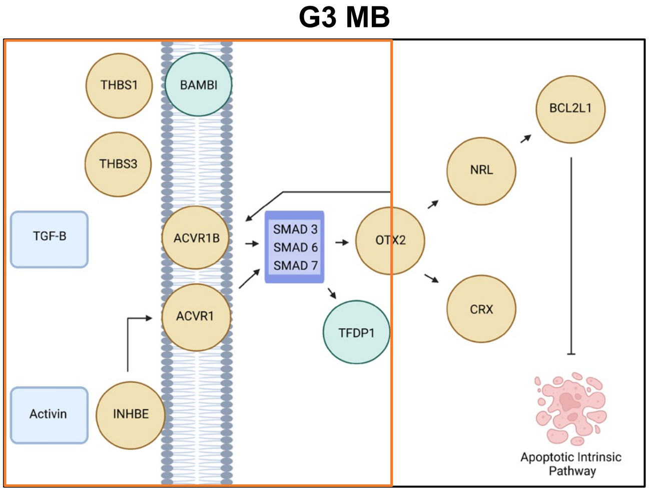
G3 MB
THBS1
BAMBI
THBS3
TGF-B
ACVR1B
SMAD 3
SMAD 6
SMAD 7
OTX2
NRL
BCL2L1
CRX
TFDP1
ACVR1
Activin
INHBE
Apoptotic Intrinsic
Pathway

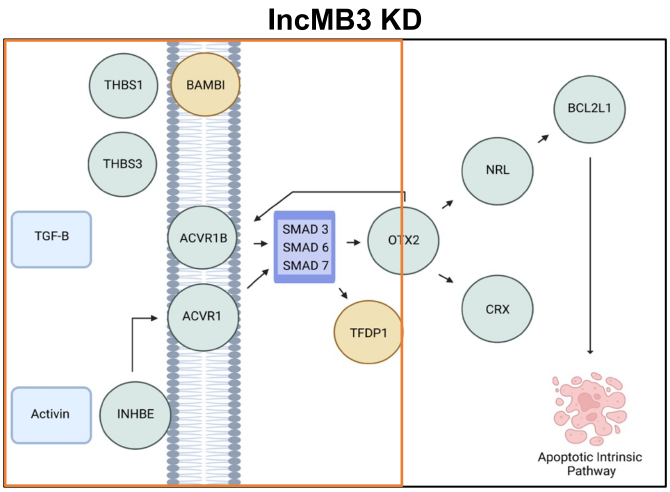
lncMB3 KD
THBS1
BAMBI
THBS3
TGF-B
ACVR1B
SMAD 3
SMAD 6
SMAD 7
OTX2
NRL
BCL2L1
CRX
TFDP1
ACVR1
Activin
INHBE
Apoptotic Intrinsic
Pathway

**Figure S3. RNA-Seq data mining – Referred to Figure 1**

**A.** qRT-PCR validation of RNA-Seq data in gap_*lncMB3* compared to gap_SCR condition, set as 1. Validated candidates (10 RNAs) are indicated in histograms. Data (means ± SEM) are expressed in arbitrary units and are relative to *GAPDH* mRNA levels. $N = 3$, * $p \leq 0.05$, ** $p \leq 0.01$, *** $p \leq 0.001$ (two- tailed Student's *t*-test).

**B.** GO showing the distribution in clusters of the DEGs in gap_*lncMB3* *vs* gap_SCR D283 Med cells, according to RNA-Seq data: Upregulated (RED) and Downregulated (BLUE) sets referring to WikiPathways Database. Categories are listed considering $-\log_{10}$(pvalue).

**C**. GSEA showing the differentially expressed TGF-β pathway components in gap_*lncMB3* *vs* gap_SCR D283 Med cells, according to RNA-seq data.

**D.** Schematic representation of the relevant validated genes in the tumour (G3 MB, left panel) and in the lncMB3 KD condition (right panel). Orange squares highlight the TGF-β pathway components.

S4A

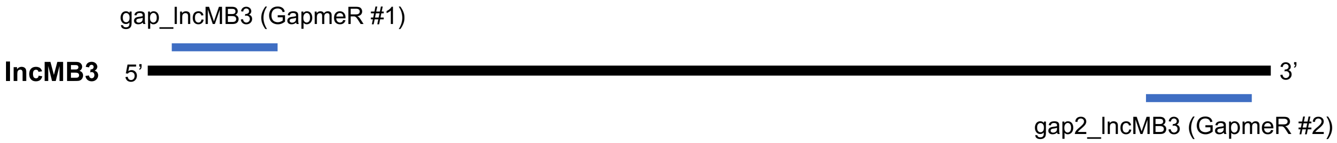
gap_lncMB3 (GapmeR #1)
lncMB3
5’
3’
gap2_lncMB3 (GapmeR #2)

S4B

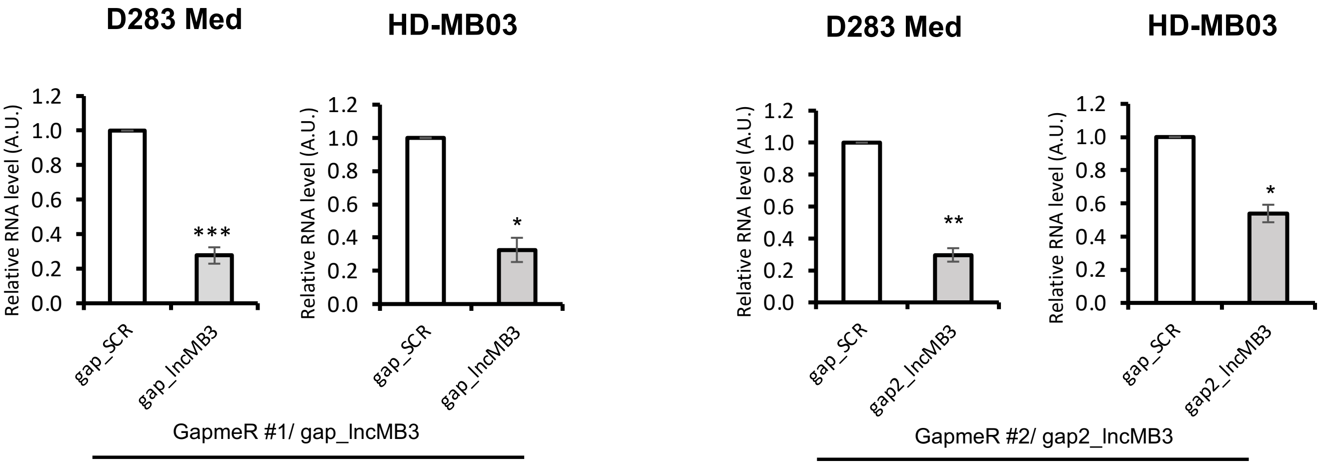
D283 Med
HD-MB03
D283 Med
HD-MB03
Relative RNA level (A.U.)
1.2
1.0
0.8
0.6
0.4
0.2
0.0
***
*
**
*
gap_SCR
gap_lncMB3
gap2_lncMB3
GapmeR #1/ gap_lncMB3
GapmeR #2/ gap2_lncMB3

S4C

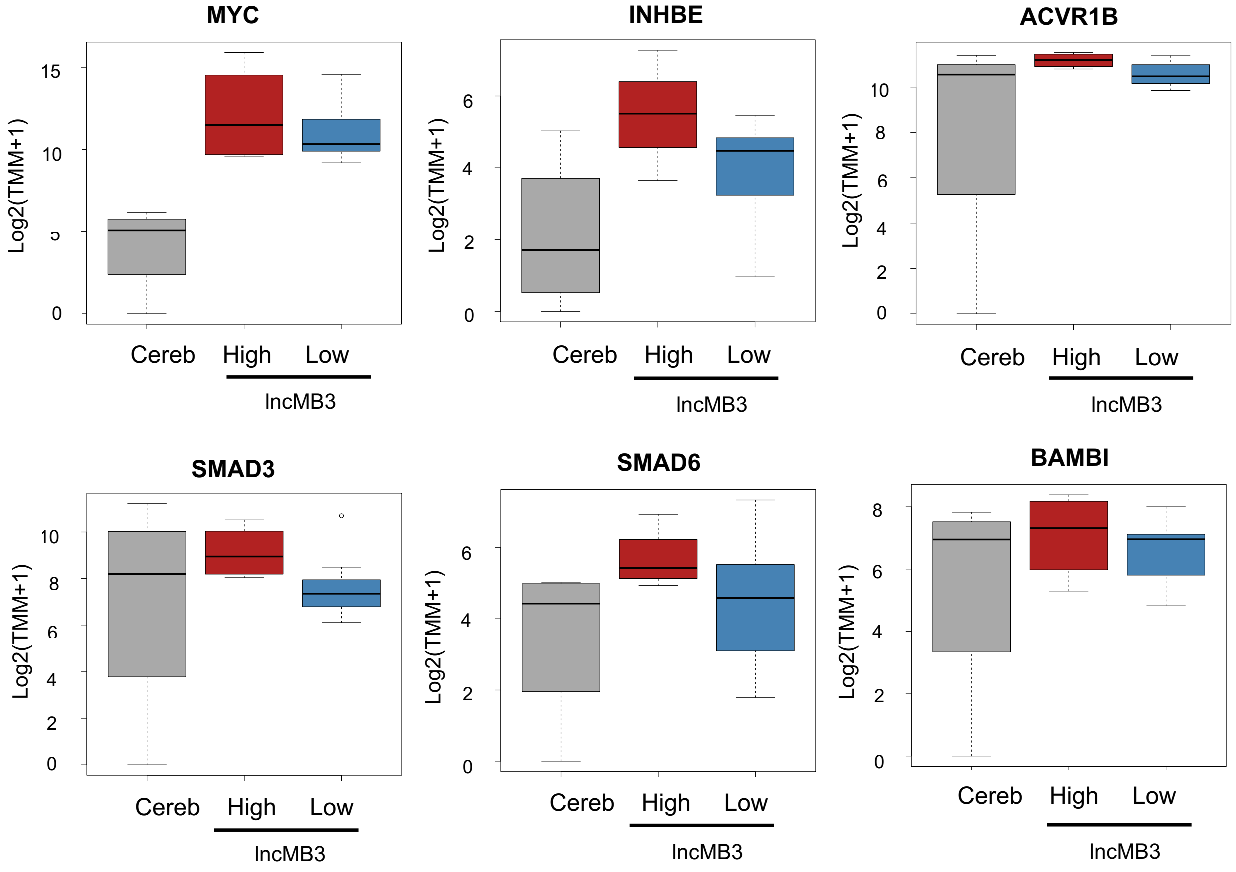
MYC
INHBE
ACVR1B
SMAD3
SMAD6
BAMBI
Log2(TMM+1)
Cereb
High
Low
lncMB3

S4D

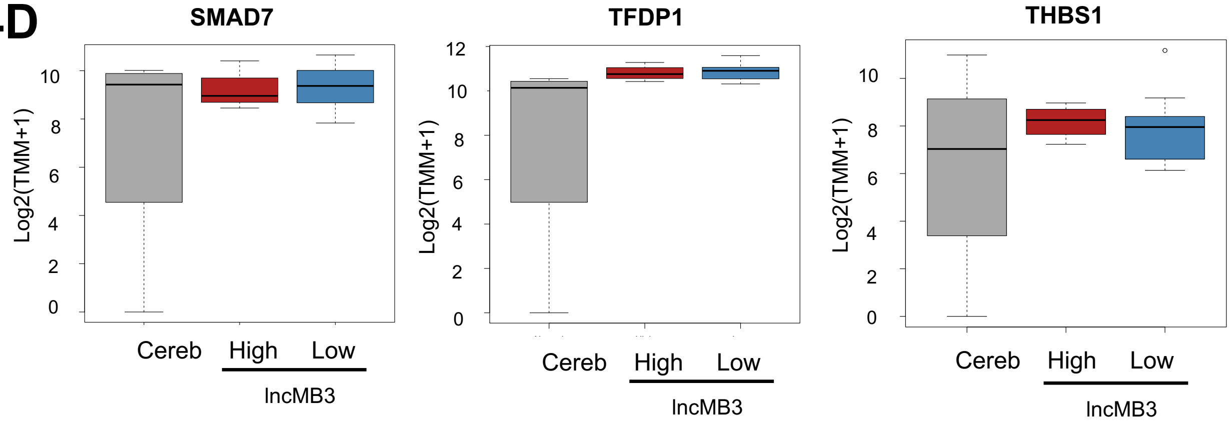
SMAD7
TFDP1
THBS1
Log2(TMM+1)
Cereb
High
Low
lncMB3

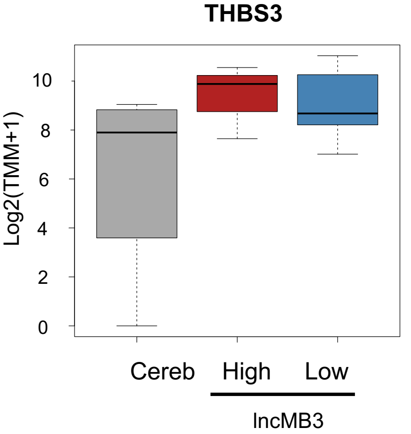
THBS3
Log2(TMM+1)
Cereb
High
Low
lncMB3

S4E

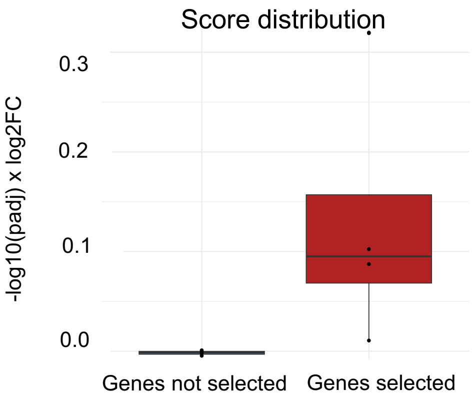
Score distribution
-log10(padj) x log2FC
0.0
0.1
0.2
0.3
Genes not selected
Genes selected

**Figure S4. Validation of *lncMB3* KD specificity – Referred to Figure 1, 2 and 3**

**A.** Schematic representation of GapmeR #1 and GapmeR #2 (blue lines) binding to *lncMB3* (black line).

**B.** Left Panels: qRT-PCR analysis of *lncMB3* in D283 Med and HD-MB03 cells upon *lncMB3* KD (GapmeR #1). Right Panels: qRT-PCR analysis of *lncMB3* D283 Med and HD-MB03 cells upon *lncMB3* KD (GapmeR #2). $N = 3$, * $p \leq 0.05$, ** $p \leq 0.01$, *** $p \leq 0.001$ (two- tailed Student's).

**C.** Boxplots showing the expression levels ($\log_2$(TMM+1)) of selected *lncMB3*-associated genes/targets (MYC, INHBE, ACVR1B, SMAD3, SMAD6, and BAMBI, indicated above each diagram) across three sample groups from dataset GSE164677: healthy cerebellum (Cereb), G3 MB patients with high (High) or low/negative (Low) lncMB3 expression.

**D.** Boxplots showing the expression levels ($\log_2$(TMM+1)) of *lncMB3* non-validated targets (SMAD7, TFDP1, THBS1, and THBS3). Details as in Panel C.

**E.** Wilcoxon rank-sum test showing the distribution of the composite differential expression score (–log10(padj) × log2FoldChange) for *lncMB3* target genes (*INHBE, ACVR1B, SMAD3, SMAD6*) *vs* non-target genes (*SMAD7, THBS1, THBS3, TFDP1*) in Group 3 MB samples.

**S5A**

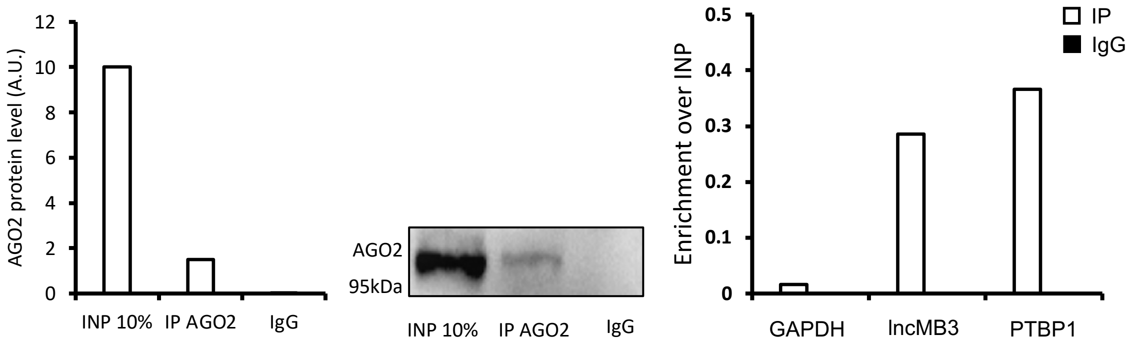


**Figure S5. AGO2 CLIP assay in D283 Med cells – Referred to Figure 4**

**A.** Left Panel: Western blot analysis of AGO2 levels in AGO2- (IP), IgG-immunoprecipitated cell extracts, and in Input sample, as control. Right panel: qRT-PCR analysis of RNA enrichment over Input in IP and IgG fractions. *N* = 1. Data expressed as percentage of Input. *GAPDH* and *PTBP1* mRNAs were used as negative and positive control, respectively.

# S6A

| Gene | log2FC Even VS inp | FDR even VS inp | log2FC odd VS inp | FDR odd VS inp |
|---|---|---|---|---|
| lncMB3 | 9.523853 | 0.007245 | 7.941424 | 0.028996 |
| HMGN5 | 6.060362 | 0.00112745 | 7.475088 | 0.004114 |
| ANKDD1A | 7.0368 | 0.011791 | 6.388846 | 0.029212 |
| EIF5B | 4.852299 | 0.017849 | 5.803747 | 0.009553 |

# S6B

HMGN5 Expression from GTEX (Release V6)

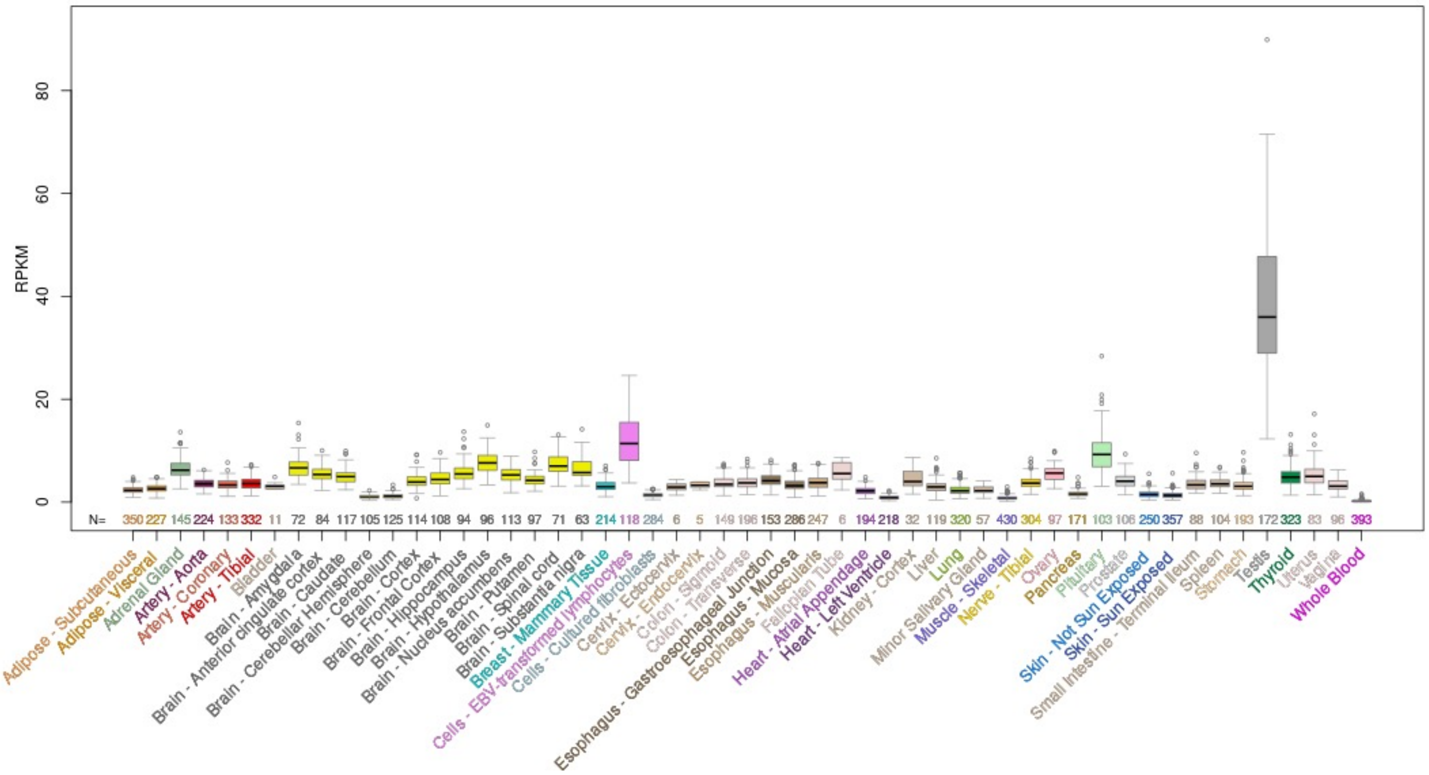


# S6C

| SAMPLE | TARGET | COPY NUMBER |
|---|---|---|
| D283 Med | LncMB3 | 135 |
| D283 Med | HMGN5 | 213.9 |
| D283 Med | GAPDH | 2051 |

**Figure S6. Analysis of *lncMB3* mechanism of action – Referred to Figure 4**

**A.** Table showing the genes enriched according to the RNA-Seq analysis of two independent native *lncMB3* pull-down assays performed in total extracts of D283 Med cells.

**B.** Expression of *HMGN5* from 54 healthy tissues, according to GTEx Analysis, Release V6. Levels are shown in RPKM. Circles represent outliers. Details at https://gtexportal.org/home/gene/HMGN5

**C.** Table showing the absolute number of molecules per µL for *lncMB3*, *HMGN5* and *GAPDH*, according to the evaluation performed through digital PCR analysis.

**S7A**

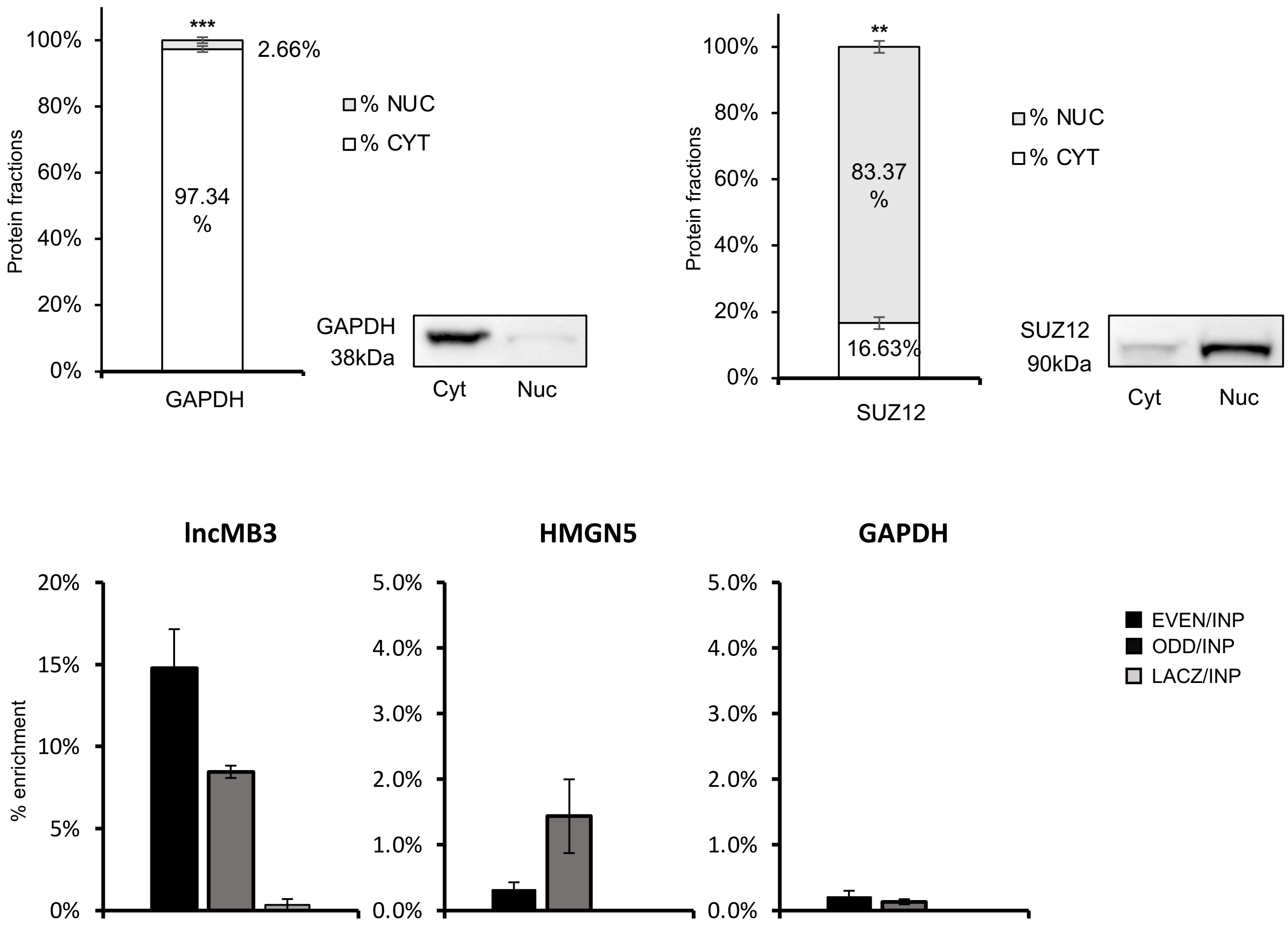


**Figure S7. Analysis of *lncMB3* mechanism of action – Referred to Figure 4**

**A.** Upper Panel: the efficiency of nucleus/cytoplasm fractionation in D283 Med cells was assessed by evaluating GAPDH and SUZ12 levels, respectively. Normalisation was performed on total protein quantity. Data (means ± SEM) are expressed as percentage of total GAPDH or SUZ12 levels. *N* = 3. Lower Panel: qRT-PCR analysis of *lncMB3* and *HMGN5* RNA enrichment in EVEN, ODD and LacZ fractions over Input, from native *lncMB3* RNA pull-down experiments of D283 Med cell cytoplasmic extracts. Data expressed as percentage of Input, *N* = 3.

**S8A**

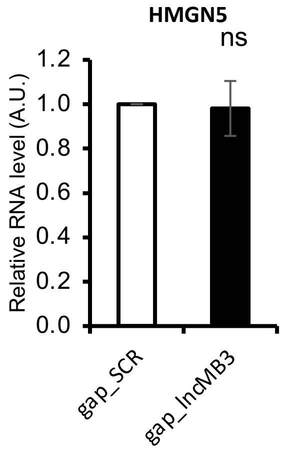


**S8B**

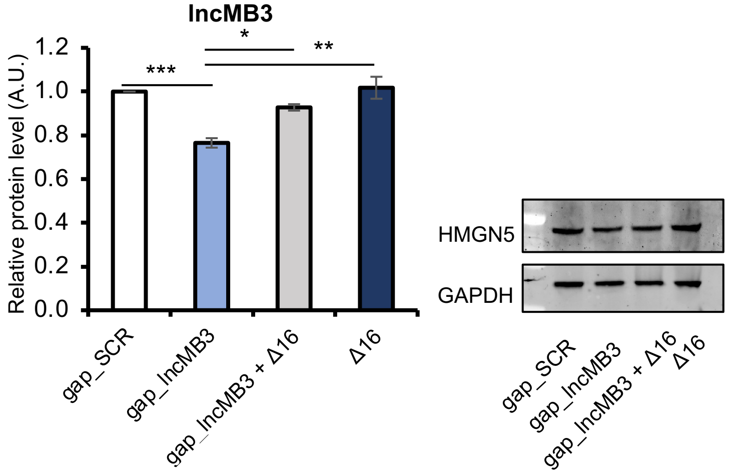


**S8C**

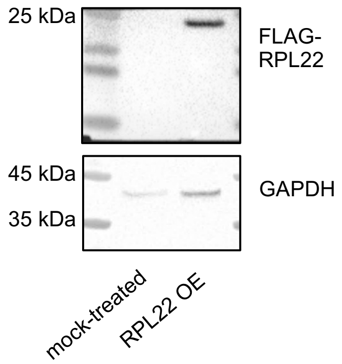


**S8D**

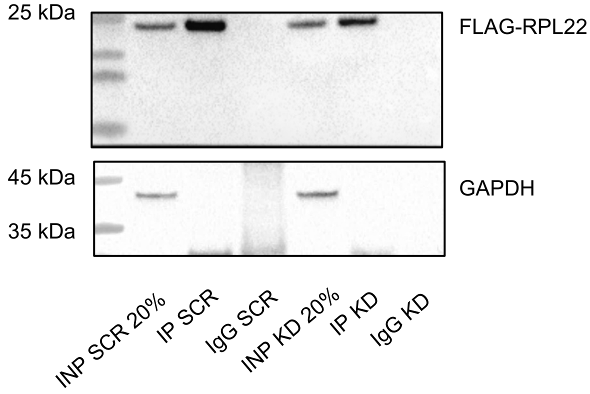


**S8E**

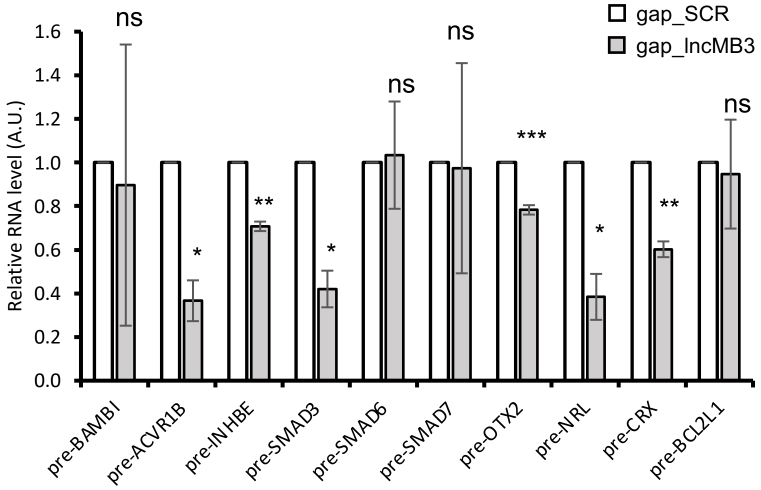


**S8F**

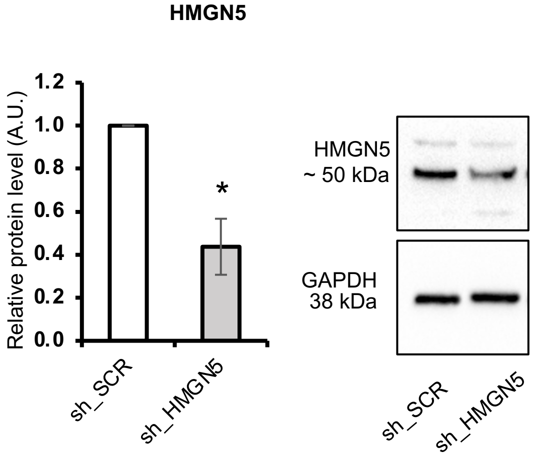


**S8G**

| Gene Name | KD | % downregulation |
|---|---|---|
| INHBE | gap_lncMB3 | ~80% |
| | sh_HMGN5 | ~30% |
| SMAD3 | gap_lncMB3 | ~80% |
| | sh_HMGN5 | ~50% |
| OTX2 | gap_lncMB3 | ~80% |
| | sh_HMGN5 | ~20% |
| BCL2L1 | gap_lncMB3 | ~40% |
| | sh_HMGN5 | ~30% |

**Figure S8. Analysis of *lncMB3* mechanism of action – Referred to Figure 4**

**A.** qRT-PCR analysis of *HMGN5* mRNA in D283 Med cells upon *lncMB3* KD (GapmeR #1). Data (means ± SEM) are expressed in arbitrary units and are relative to *GAPDH* mRNA levels. *N* = 3, (two- tailed Student's *t*-test).

**B.** Western blot analysis of *lncMB3* expression in D283 Med cells transfected with 1 pulse of gap_SCR, gap_*lncMB3*, gap_*lncMB3* + Δ16 construct or Δ16 construct alone. *N* = 3. * $p \leq 0.05$, ** $p \leq 0.01$, *** $p \leq 0.001$ (two- tailed Student's).

**C.** Western blot analysis of RPL22 protein levels in mock-treated or FLAG-RPL22 construct overexpressing D283 Med cells. Normalisations were performed relative to GAPDH protein levels. *N* = 1.

**D.** Western blot analysis of RPL22 protein levels in fractions (Inp, IP, IgG) from RIP assay performed in gap_SCR, or gap_*lncMB3* conditions. Normalisations were performed relative to GAPDH protein levels. *N* = 1.

**E.** qRT-PCR analysis of pre-mRNAs of the TGF-β and following pathways upon *lncMB3* KD (GapmeR #1) relative to gap_SCR, set as 1. Data (means ± SEM) are expressed in arbitrary units and are relative to *GAPDH* mRNA levels. *N* = 3, * $p \leq 0.05$, ** $p \leq 0.01$, *** $p \leq 0.001$ (two- tailed Student's *t*-test).

**F.** Western blot analysis of HMGN5 protein levels in D283 Med cells treated with sh_SCR or sh_HMGN5 after 72 hours. Normalisations were performed relative to GAPDH protein levels. *N* = 3, * $p \leq 0.05$ (two- tailed Student's *t*-test).

**G.** Table showing the % of downregulation of *INHBE, SMAD3, OTX2 and BCL2L1* upon gap_*lncMB3* or sh_HMGN5 in D283 Med cells.

**S9A**

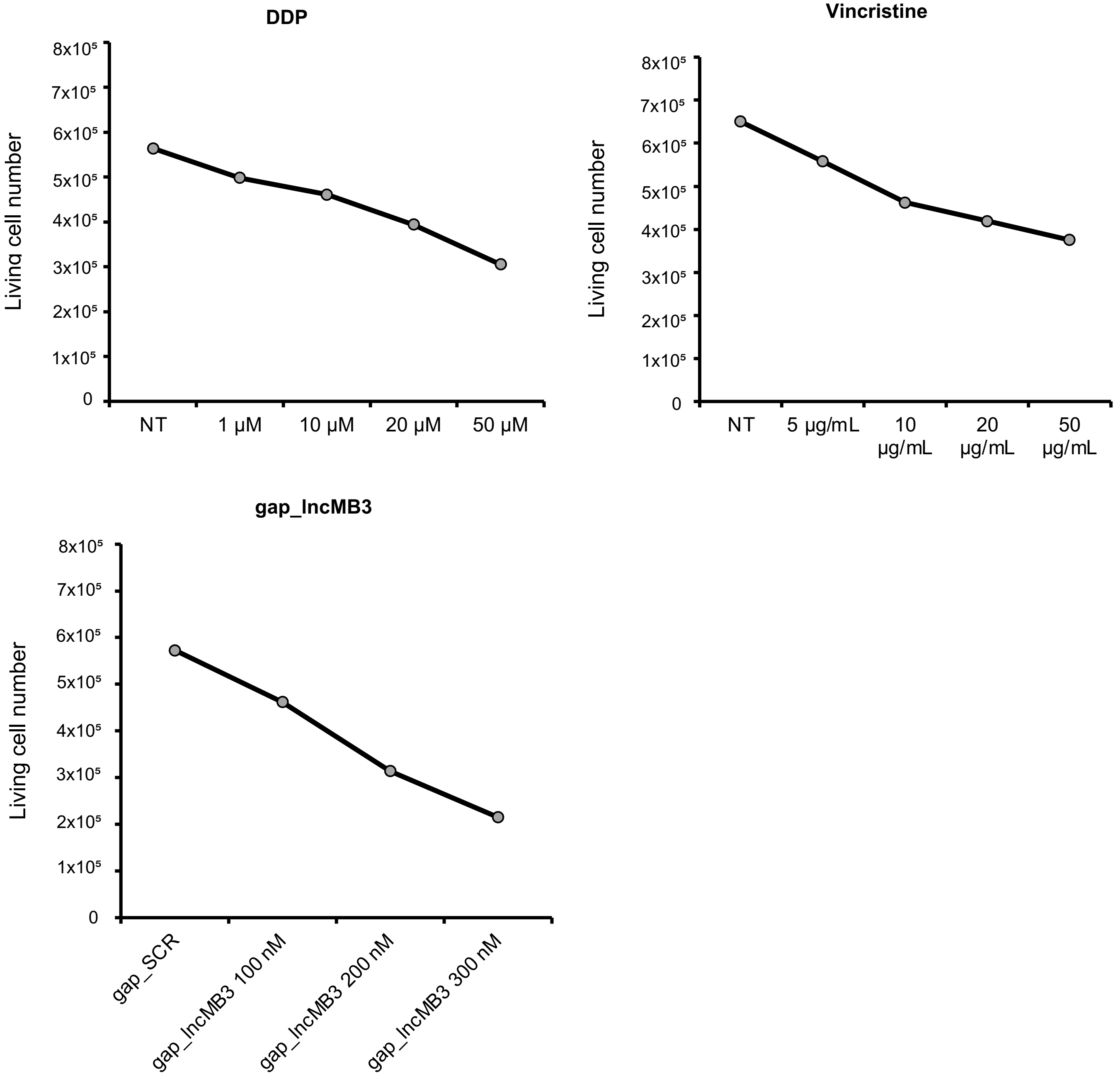
DDP
Living cell number
8x10⁵
7x10⁵
6x10⁵
5x10⁵
4x10⁵
3x10⁵
2x10⁵
1x10⁵
0
NT
1 µM
10 µM
20 µM
50 µM
Vincristine
Living cell number
NT
5 µg/mL
10 µg/mL
20 µg/mL
50 µg/mL
gap_lncMB3
Living cell number
gap_SCR
gap_lncMB3 100 nM
gap_lncMB3 200 nM
gap_lncMB3 300 nM

**S9B**

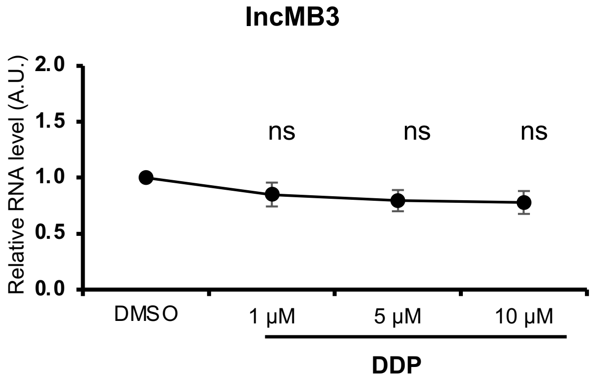
lncMB3
Relative RNA level (A.U.)
2.0
1.5
1.0
0.5
0.0
ns
ns
ns
DMSO
1 µM
5 µM
10 µM
DDP

# S9C

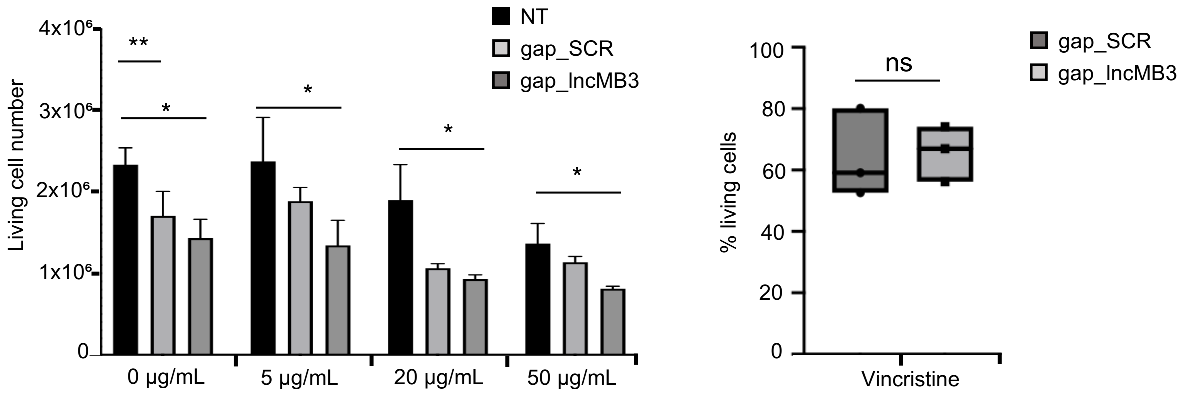


# S9D

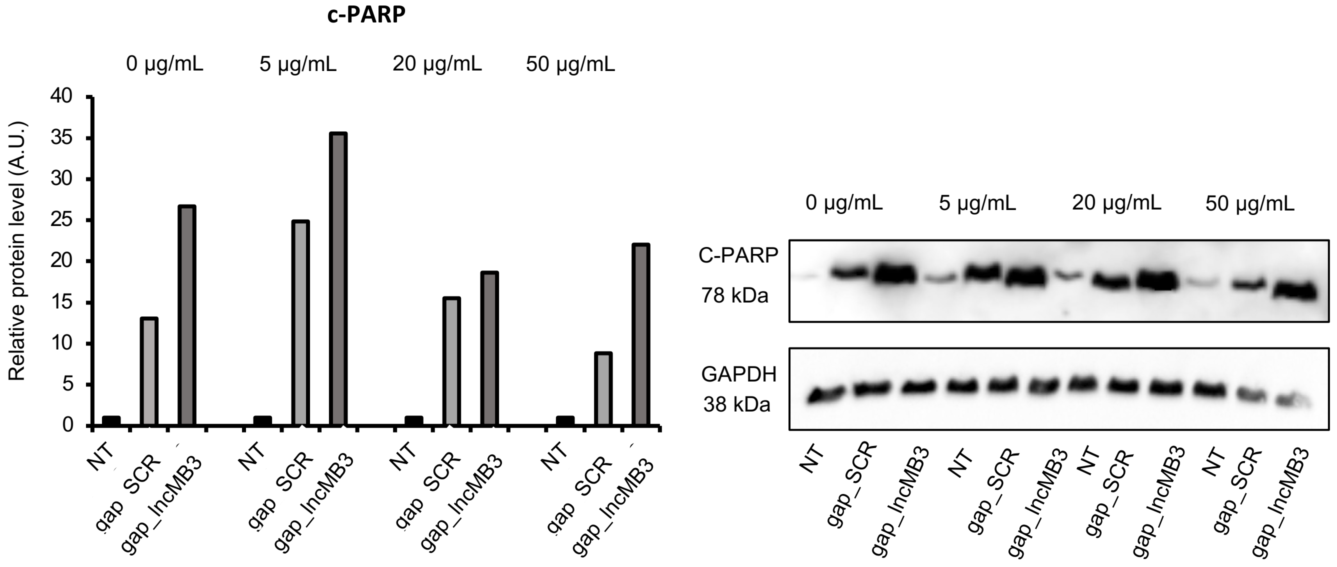


# S9E

| Drug | D1 (chemo IC50, combo) | D2 (GapmeR IC50, combo) | Dx1 (chemo IC50, alone) | Dx2 (GapmeR IC50, alone) | CI |
|---|---|---|---|---|---|
| DDP | 10 µM | - | 50 µM | - | 0.7 |
| Vincristine | 20 µg/mL | - | 50 µg/mL | - | 0.9 |
| GapmeR | | 100 nM | | 200 nM | |

**Figure S9. Analysis of *lncMB3* KD/chemotherapeutic treatments on D283 Med cells – Referred to Figure 5**

**A.** Dose-response analysis of the number of viable D283 Med cells upon different treatments, indicated above. Control cells (NT) were treated with the vehicle (DMSO). Cell counts were conducted 24 hours after drug administration. *N* = 1.

**B.** qRT-PCR analysis of *lncMB3* in D283 Med cells treated for 24 hours with DDP at different doses (reported on the x-axis). Control cells were treated with vehicle (DMSO) and set as 1. Data (means ± SEM) are expressed in arbitrary units and are relative to *GAPDH* mRNA levels. *N* = 3, (two- tailed Student's *t*-test).

**C.** Left panel: dose-response analysis of the number of viable D283 Med cells upon Vincristine treatment. Cells were treated with the vincristine vehicle (DMSO), 5, 20 or 50μg/mL vincristine, untreated (NT) or treated with gap_SCR or gap_*lncMB3* (GapmeR #1). Cell counts were conducted 48 hours after vincristine/GapmeR treatments. *N* = 3, * $p \leq 0.05$, ** $p \leq 0.01$ (two- tailed Student's). Right panel: double-treated samples (gap_SCR + Vincristine and gap_*lncMB3* + Vincristine) were normalised on the viable cell number of the corresponding transfected-only sample (gap_SCR and gap_*lncMB3*, respectively). Vincristine concentration was 20μg/mL. *N* = 3, (two- tailed Student's t-test).

**D.** Left panel: quantification of c-PARP protein levels in D283 Med cells upon Vincristine treatment in untreated (NT), gap_SCR or gap_*lncMB3* samples, in the same conditions as in panel B. GAPDH was used as control. Right panel: Western blot analysis of c-PARP protein levels from the experiment described in left panel. Normalisation was performed on GAPDH protein levels. *N* = 1 for each condition (drug concentration).

**E.** Table showing drug concentrations employed to calculate the CI values, based on the experiments in Fig. S9A, 5A, S9C.

**S10A**

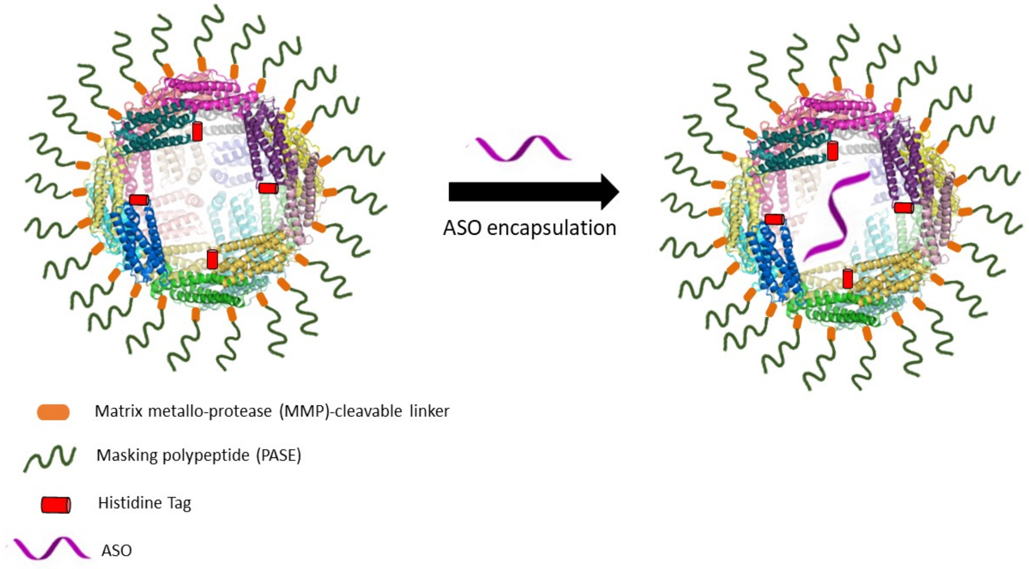


**S10B**

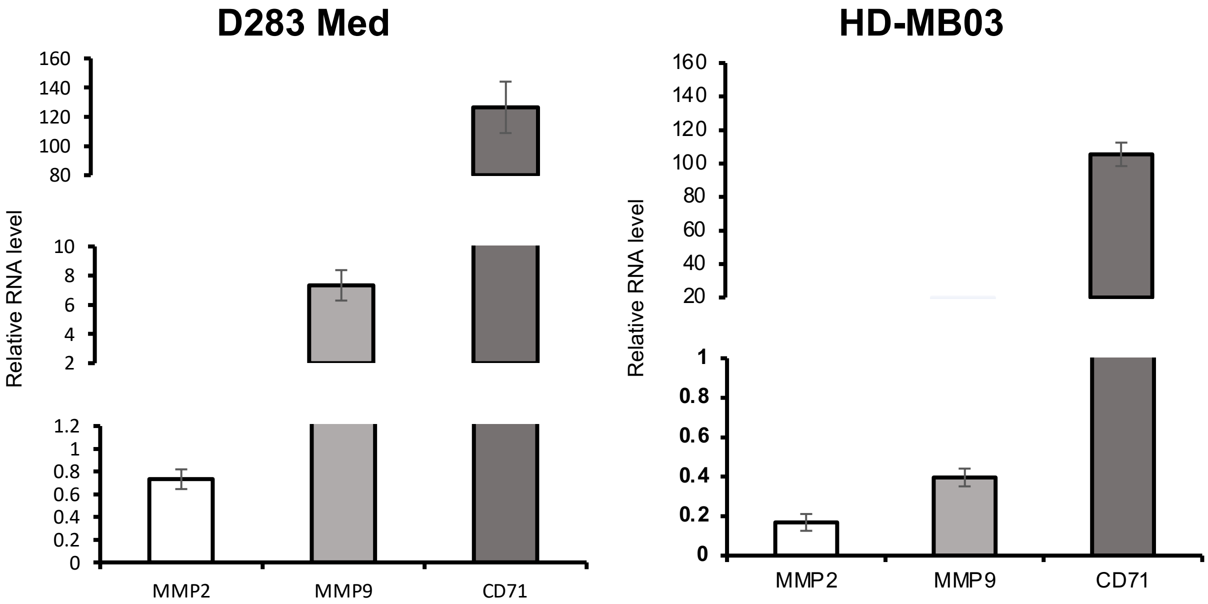


**S10C**

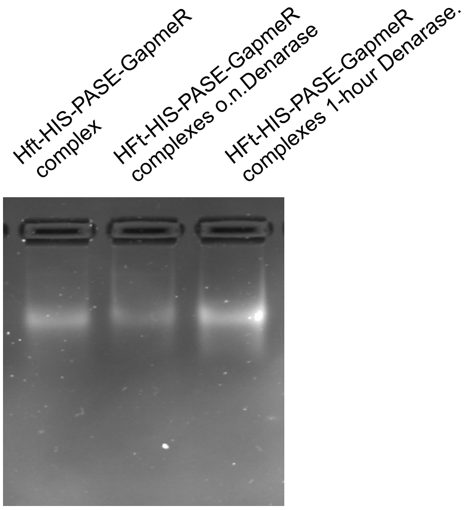


**S10D**

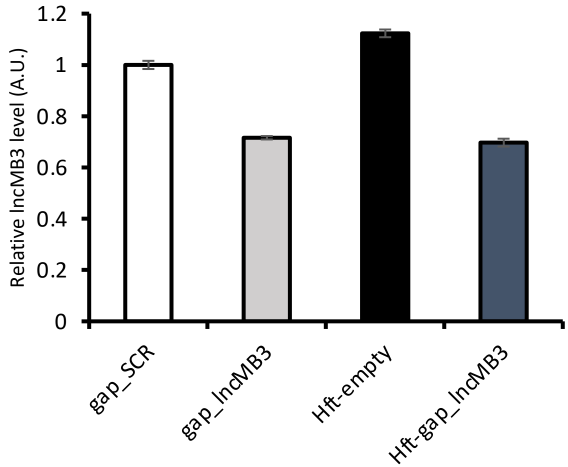

**Figure S10 – Molecular analysis related to HFt-HIS-PASE-GapmeR complex delivery – Referred to Figure 6**

**A.** Schematic representation of HFt-HIS-PASE and HFt-HIS-PASE-GapmeR encapsulation in the protein cavity. Single domains and molecules are described in the legend. To allow the internal surface of the protein to be visualised (lighter colours), only 18 monomers out of the 24 are shown. In addition, for clarity purposes, only 4 out of the 24 histidine tags are shown. The picture has been generated with Pymol and GNU Image Manipulation Programs.

**B.** qRT-PCR analysis of *CD71*, *MMP 2* and *MMP 9* mRNA levels in D283 Med (left panel) or HD-MB03 (right panel) cells. Data (means ± SEM) are expressed in arbitrary units and are relative to *GAPDH* mRNA levels.

**C.** HFt-HIS-PASE-GapmeR complexes before and after Denarase treatment at 37°C visualised as band migration profiles on agarose gel electrophoresis. *Lane 1:* HFt-HIS-PASE-GapmeR complex; *Lane 2:* HFt-HIS-PASE-GapmeR complexes after overnight incubation with Denarase; *Lane 3*: HFt-HIS-PASE-GapmeR complexes after 1-hour incubation with Denarase. Gel was stained with SYBR Gold.

**D.** qRT-PCR analysis of *lncMB3* levels upon KD with GapmeR #1 (gap_*lncMB3*) or HFt-HIS-PASE-GapmeR complexes (HFt-gap_*lncMB3*), relative to gap_SCR, set as 1. Data are expressed in arbitrary units and are relative to *GAPDH* mRNA levels. *N* = 1.

Where necessary in the figure HFt-HIS-PASE is referred as HFt.
