# Supplementary material for: The MYC-dependent lncRNA MB3 inhibits apoptosis in Group 3 Medulloblastoma by regulating the TGF-β pathway via HMGN5
Source: Cell Death Dis. 2025 Nov 6;16(1):800. doi: 10.1038/s41419-025-08097-8 (PMC12592558; doi:10.1038/s41419-025-08097-8)
Supplement: Supplementary file 2 — Supplementary Materials and Methods [file 41419_2025_8097_MOESM2_ESM.pdf]

SUPPLEMENTARY MATERIALS AND METHODS

Cell Cultures

All cell lines were sorced from ATCC. Semi-adherent D283 Med cells were cultured in MEM medium (M2279, Sigma-Aldrich) supplemented with 20% of heat-inactivated foetal bovine serum (USA origin), 1% Sodium Pyruvate, 1% MEM Non-Essential Amino Acids Solution, 1% Glutamax, and 1x Penicillin/Streptomycin. Adherent HD-MB03 cells were cultured in RPMI medium (R0883, Sigma-Aldrich) supplemented with 10% of heat-inactivated foetal bovine serum (USA origin) and 1x Penicillin/Streptomycin.

Cell Propagation

Floating D283 Med cell subpopulation was collected, while adherent cell fraction was washed with 1x PBS w/o Ca^{++}/Mg^{++} and detached using 1x Trypsin/EDTA (T4299, Sigma-Aldrich) for 5 min. Cells were centrifuged at 800 RPM for 5 min and resuspended in fresh culture medium. Adherent HD-MB03 cells were processed similarly. Cells were counted on a CytoSMART Cell Counter (CLS6749, Corning, Sigma-Aldrich) and replated at the desired concentration.

Evaluation of Viable Cell Number

D283 Med cells and HD-MB03 cells were seeded in plates and treated as per experimental plans (GapmeR/plasmid transfection and/or chemotherapeutic treatment), with counts taken at specified time points. Specifically, 10µL cell aliquots were thoroughly suspended, mixed to 10µL of trypan blue and quantified by CytoSMART Cell Counter.

Plasmids construction

Δ16: D283 Med cell cDNA was used as template to amplify a mutant version of *lncMB3* sequence lacking GapmeR #1 terminal binding site with DNA oligos MB3 FW and MB3 REV. The EPB-PURO-TT backbone was linearised with Delta16 FW - T7 REV oligos. Construct was generated using In-Fusion Snap Assembly Cloning Kit (Takara Bio).

sh_HMGN5: the EPB-BSD-PGK backbone was linearized with oligos shRNA_TOOL_fw and shRNA_TOOL_rev and manipulated according to In-Fusion Snap Assembly Cloning Kit (Takara Bio). Control plasmid was obtained by (29).

sh_HMGN5 sequence: GTTGTTGAAGAAGACTACAAT

Transfections

Negative control (LG00000002, Qiagen) or specific LNA GapmeRs were transfected 1-3 times at 24 hours intervals in D283 Med or HD-MB03 cells (100 nM) with Lipofectamine 2000 (11668-019, Invitrogen, ThermoFisher Scientific), in opti-MEM medium (31985070, Gibco, ThermoFisher Scientific), following manufacturer's instructions.

Control (empty EPB-PURO-TT) and Δ16 plasmids were transfected in D283 Med cells (100 ng) as described above. Doxycycline (50-100 ng/mL, D9891, Sigma Aldrich, Merck KGaA) was added in the complete growing medium.

Five µg of sh_SCR (negative control) or sh_HMGN5 plasmids were transfected twice (24 and 48 hours after cell seeding) in D283 Med cells as already described.

RNA Extraction and Analysis by qRT-PCR

Total RNA was extracted by Direct-zol RNA MiniPrep (R2052, Zymo Research). cDNA synthesis for quantitative real-time PCR (qRT-PCR) assay was performed by Takara PrimeScript RT Reagent Kit (RR037A, Takara-bio). qRT-PCR was carried out using the SensiFAST SYBR Lo-ROX

Kit (BIO-94020, Bioline) on a 7500 Fast Real-Time PCR (Applied Biosystem), with *GAPDH* or *ATP5O* as a reference target.

Digital PCR analysis

Digital PCR analysis was performed on the QIAcuity® nanoplate digital PCR 8.5K 24-well (Qiagen). The reaction mix was prepared as per the manufacturer's instructions. Twelve µl of PCR mix were loaded per nanoplate well, and automatically partitioned in 8500 sections. EVA green signal acquisition used a 500 ms exposure duration of and a gain of 6.

Immunoblotting

Proteins were extracted from MB cells in RIPA Buffer (50 mM Tris–HCl (pH 8), 150 mM EGTA, 150 mM NaCl, 50 mM NaF, 10% glycerol, 1.5 mM $MgCl_2$, 1% Triton). Lysates were separated on gradient poly-acrylamide gels and transferred to Amersham Protran 0.45 µm nitrocellulose membrane (10600002, GE Healthcare Life Sciences), through the NuPAGE System (EI0002, Invitrogen). Immunoblotting involved the following antibodies: anti-GAPDH (sc-32233, Santa Cruz Biotechnology); anti-FLAG (F1804, Sigma-Aldrich); anti-cleaved PARP-1 (bsm-52408R, Bioss); anti-PARP-1 (PA5-34803); anti-HMGN5 (720391, Invitrogen); anti-OTX2 (ab183951, Abcam); anti-BCL2L1 (2764S, Cell Signaling); anti-ATP5O (A305-419A, Bethyl Laboratories). Protein staining was performed by WesternBright ECL (K-12045-D50, Advansta), detected by ChemiDoc XRS+ Molecular Imager (Bio-Rad) and quantified through the Image Lab Software (release 3.0.1).

RNA-Seq and differential expression analysis

Sequencing libraries from polyA+ RNA extracted from D283 Med cells (3 replicates) were prepared using the TruSeq Stranded mRNA Library Prep Kit (Illumina) and sequenced on a Novaseq 6000 system with 100-bp paired-end reads (>20M depth). Trim Galore188 (v0.6.4_dev) was used to remove adapters and low-quality bases; a minimum read length of 20 was enforced. STAR (v2.7.9a) aligned reads to the GRCh38 genome, and quality was assessed using FastQC. Unique mapping exceeded 85%. The "--quantMode TranscriptomeSAM" option was used to generate alignments translated into transcript coordinates. Transcript and gene quantification was performed using RSEM. Differential expression analysis (DEA) used the Bioconductor DESeq2 package, filtering for DEA the genes with absolute expression of at least 10 in three of six samples. Shrinked logFC was calculated using "lfcShrink" function. FDR < 0.05 and |logFC| > 0.6 were criteria for considered differentially expressed genes.

Gene Ontology

Gene Ontology (GO) analysis was assessed using the DAVID tool (https://david.ncifcrf.gov/tools.jsp) on Wikipathways databases for both significantly upregulated and downregulated genes.

Gene Set Enrichment Analysis

Gene Set Enrichment Analysis (GSEA) employed the Bioconductor package "fgsea" with the whole gene set of the experiment employed, specifically, logFC and padj values. Genes were ranked in a sample dataset by multiplying $-\log_{10}$(FDR) * logFC according to their differential expression between two experimental groups. Ensemble Gene IDs were mapped to Entrez Gene IDs using BioMart, and the "fgsea" function was run with minSize = 15, maxSize = 500

and nPermSimple = 10000. The analysis was performed on the gene sets belonging to the Molecular Signatures Database (MsigDb v7.4). For each gene set, an enrichment score was computed and assigned to the gene set of the ranked gene lists. Additionally, GSEA calculated a normalised enrichment score to account for variations in pathway size, ensuring robustness across different gene sets and datasets. An FDR of 25% was used for this analysis.

Dataset reanalysis

RNA-Seq data from GSE164677, were reanalysed following the pipeline described in (10).

Briefly, raw sequencing reads were first subjected to adapter trimming and quality filtering using Trim Galore (https://www.bioinformatics.babraham.ac.uk/projects/trim_galore/, accessed on 23 September 2019). Normalization of library sizes was conducted via the normLibSizes() function in edgeR, which applies Trimmed Mean of M-values (TMM) normalization by default. Genes were considered differentially expressed if they met the criteria of false discovery rate (FDR) < 0.001 and absolute log2foldchange > 2. G3 MB samples were stratified according to *lncMB3* High vs Low expression. For data visualization, boxplots were generated using log2-transformed TMM values (log2(TMM + 1). Gene distribution scores were assessed by Wilcoxon rank-sum test.

Treatments with chemotherapeutics

Chemotherapeutics (cisplatin, HY-17394, MedChem; vincristine HY-N0488, MedChem) were resuspended in DMSO and administered as per experimental protocol. For combined treatments, the selected drug amount was administered 5 hours post-transfections. Cells count/analysis occurred 24/48 hours after treatment. Combinatorial Index (CI) was calculated according to the Chou-Talalay method [38].

Flow Cytometry Analyses

Apoptosis induction was analysed by flow cytometry determination of Annexin V-FITC staining (Enzo Life Sciences, ALX-850-020), to discriminate viable (FITC-/PI-), early apoptotic (FITC+/PI-) and late or necrotic cells (FITC+/PI+). Cells were collected and resuspended in binding buffer containing Annexin V-FITC and incubated at RT for 15 min. PI was added before reading the sample to flow cytometer (BD FACSCalibur; Beckman Coulter - CytoFLEX SRT). Data were analysed using the Flowing software. Ten thousand events were collected from each sample.

Nucleus/cytoplasm separation

Nucleus/cytoplasm separation was performed on D283 Med cells as follows. Cells were washed with PBS (without Ca2+ and Mg2+) and lysed in buffer A (Tris 20 mM pH 8.0, NaCl 10 mM, MgCl2 3 mM, NP40 (IGEPAL) 0.10%, EDTA 0.2 mM, DTT 1 mM, Protease inhibitor cocktail 1×, Ribolock 1×), and incubated on ice for 5 min. Then samples were centrifuged 5 min 1000 RPM 4°C and cytoplasmic fraction was collected. Then pellets were washed using buffer A and centrifuged 5 min 1000 RPM 4°C. Supernatant was discarded and nuclei were incubated in buffer C (tris 20mM pH 8.0, NaCl 400mM, Glycerol 20%, DTT 1 mM, Protease inhibitor cocktail 1×, Ribolock 1×) in ice for 5 min. Samples were then homogenised and analysed via immunoblotting**.** Cytoplasmic RNA pull-down assays were conducted using 1mg of cytoplasmic cell extract of D283 Med cells per sample, and performed as described for native RNA pull-down assays.

Native RNA pull-down assay

Native RNA pulldown experiments were performed as described in (28). Briefly, 1x10^8 proliferating D283 Med cells were centrifuged at 400 g for 5 min and pellets were lysed for 30 min on rotating wheel at 4°C in Cell Lysis Buffer (Tris-HCl pH 7,5 50 mM, NaCl 150 mM, MgCl2 3 mM, NP40 0.5%, EDTA 2 mM, DTT 1 mM; 1× PIC and RNase inhibitors). The lysate was cleared by centrifugation at 15000 g for 15 min at 4°C. One mg of supernatant (whole cell extract) was diluted in a 1:2 ratio with Hybridization Buffer (Tris-HCl pH 7.5 100 mM, NaCl 300 mM, MgCl2 1 mM, SDS 0.2%, Formamide 15%, NP40 0.5%, EDTA 10 mM, DTT 1 mM, 1× PIC and RNase inhibitor).
10% of the total extract was collected for the Input sample. 100 pmol of biotinylated probes (EVEN, ODD and LacZ sets) were heated at 80°C for 3 minutes and added to the mix. After 4-hour incubation on rotating wheel at 4°C, 0.1 ml of washed streptavidin Magnasphere paramagnetic beads (Promega) were added, and the mixture was incubated for as before. After pull-down, beads were collected using a magnetic rack and washed 4 times with Hybridization Buffer. Then 500 µL triZOL (ThermoFisher Scientific) were added to each sample and RNA was extracted (RNeasy Mini Kit Qiagen), DNase-treated and retro transcribed using SuperScript Vilo cDNA Synthesis Kit (ThermoFisher Scientific). Pull-down qRT-PCR results were represented as a percentage of enrichment related to INP.

AMT-crosslinked RNA pull-down assay

AMT-crosslinked RNA pull-down experiments were performed as described in (28). Briefly, 1x10^8 D283 Med cells were centrifuged at 400 g for 5 min, resuspended in 10 mL of ice-cold complete PBS supplemented with 0.5 mg/ml 4'-aminomethyl-4,5',8-trimethylpsoralen (AMT, Sigma-Aldrich), and crosslinked at 365 nm five times for 2-minutes cycles. Guanidine Hydrochloride 6M was added 1:1 to AMT volume. The lysate was split into 250µL aliquots and

25µL of a 20mg/mL solution of Proteinase K (Ambion) and 6.5µL of 20% SDS were added to each aliquot and incubated at 65°C for 1 h. Then 750µL triZOL (ThermoFisher Scientific) were added to each sample and RNA was extracted (RNeasy Mini Kit Qiagen). 50µg of purified AMT-crosslinked RNA were used for each RNA pull-down sample. 500µL of RNAse-free water were added to each sample and heated at 95°C for 2 minutes and then put on ice. 1 volume of 2x Binding Buffer (containing Tris-HCl pH 7.5 20mM, NaCl 1M, EDTA 2mM, SDS 0.1%) was added to 1 volume of sample with RNAse inhibitors. 10% of the total extract was collected for the Input sample. 500 pmol of biotinylated probes (EVEN, ODD, LacZ sets) were added to the mix and samples were heated at 65°C for 3 minutes. After 4-hour incubation on rotating wheel at 4°C, 0.5ml of washed streptavidin Magnasphere paramagnetic beads (Promega) were added, and the mixture was incubated for 2h at 4°C on rotating wheel. After pull-down, beads were collected using a magnetic rack and washed 4 times with hybridization buffer. Then 500µL triZOL (ThermoFisher) were added to each sample and RNA was extracted (RNeasy Mini Kit Qiagen), UV-decrosslinked at 254nm for 10 minutes, DNase-treated and retro transcribed using SuperScript Vilo cDNA Synthesis Kit (ThermoFisher Scientific). Pull-down (PD) qRT-PCR results were represented as a percentage of enrichment related to INP.

Pull down-RNAseq and pre-processing

Raw reads in FASTQ format were retrieved from Illumina BaseSppace and adaptors removed with *fastp* tool. Clean reads were aligned to a GRCh38 assembly using STAR aligner software. Gene *loci* fragment quantification was performed on a customised gene annotation file (GTF file) based on Ensemble (release 87), using the *–quantMode GeneCounts* parameter. The GTF file was previously edited adding the *lncMB3* gene genomic coordinates and annotations. Read counts were combined into a count matrix and processed with DESeq2 R package for

normalisation and differential expression analysis. The padj threshold was set to 0,05 to consider DEGs as statistically significant.

Pull down-RNAseq analysis

edgeR was used to quantify significant genes as putative interactors on *lncMB3*, using a generalised linear model (GLM) to test for each gene. Statistically significant genes were obtained by separately comparing and intersecting EVEN and ODD samples *vs* Input samples, and then subtracting genes obtained in the comparison between LacZ *vs* Input samples. Only genes with positive FC were used and retained as putative interactors.

RNA–RNA interaction prediction

Predictions were computed using IntaRNA 3.3.2 giving as input the full FASTA sequences of transcripts of interest, using the default parameters.

microRNA *in silico* predictions

TargetScan Human database, release 8, was exploited to obtain candidates microRNA families targeting genes of interest. MicroRNA families were filtered according to conservation among vertebrates and mammals, then selected for the occurrence of conserved sites on the target transcripts, and finally filtered for those having more than -0.20 cumulative weighted context++ score, considered as the beginning of moderate confidence level of interaction. Interaction predictions were run by using a locally downloaded version of the interaction prediction tool RNA22.

Crosslinking Immunoprecipitation assay

For Crosslinking Immunoprecipitation (CLIP) assay 3.5x10^7 D283 Med cells were grown, UV-crosslinked with 0.4 J/cm2 of 254 nm UV light and collected in NP-40 lysis buffer (50 mM HEPES-KOH, 150mM KCl, 2mM EDTA, 1mM NaF, 0.5% (v/v) NP40 pH 7.4, 0.5mM DTT) by low sonication and centrifugation at 20.000 rcf. For immunoprecipitation, 2mg of cytoplasmic extracts were incubated with 10µg of AGO2 antibody or IgG as a control, and coupled to ProteinG Dynabeads resin (10004D, Invitrogen) and 10% was collected as Input. After washing with high-salt buffer (50mM HEPES-KOH, pH7,5, 500mM KCl, 0.05%(v/v) NP40, 0.5mM DTT), immunoprecipitated proteins (20% of the sample) were collected in RIPA buffer and analysed by immunoblotting. Immunoprecipitated RNA (80% of the sample) was treated by Proteinase K, extracted by Direct-zol RNA MiniPrep (Zymo Research), retro-transcribed by Superscript Vilo cDNA synthesis Kit (11754050, Invitrogen) and analysed by qRT-PCR using specific primers. RNA enrichments were reported as percentage of the input.

Ribotagging and RNA Immunoprecipitation

2x10^6 D283 Med cells were transfected with 10µg of FLAG-RPL22 expressing plasmid. On the day after, cell populations were splitted in two fractions and each sample was treated with 100nM gap_SCR or gap_*lncMB3*, respectively. 24 hours after GapmeRs administration, lysates were prepared in 350µL of POLYSOME LYSIS BUFFER (HEPES 10mM pH 7, KCl 100mM, MgCl2 5mM, NP-40 0,5%, dH20) and NT2 BUFFER, Tris 50mM pH 7.5, NaCl 150mM, MgCl2 1mM, NP-40 0.05%, dH2O). 150µL of extracts were incubated with 10µg of FLAG antibody (SLBD6976, Sigma Aldrich) or IgG (ultrapure mouse IgG, MU-003-N, Immunoreagents) as control and precipitated through ProteinG-Dynabeads resin (10004D, Invitrogen). 30µL were collected as Input. Immunoprecipitated proteins (20% of the sample) were prepared in loading dye for immunoblot. Immunoprecipitated RNA (80% of the sample) was extracted by Direct-zol RNA

MiniPrep (Zymo Research), retro-transcribed by Superscript Vilo cDNA synthesis Kit (11754050, Invitrogen) and analysed by qRT-PCR using specific primers. RNA enrichments were reported as percentage of the input.

HFt-HIS-PASE cloning, expression and purification

The design of HFt-HIS-PASE protein, a variant of the HFt-MP-PASE gene previously reported (39), includes, C- to N-terminus: (a) a first domain, comprising the amino acid sequence of the native human ferritin HFt modified through the inclusion of a 5-histidine peptide sequence, inserted in the external loop (referred as CD loop) of the ferritin surface; (b) a second N-terminal domain comprising the amino acid sequence of the matrix metalloproteinase (MMP) cleavage site (PLGLAG) followed by a Proline, Serine, Alanine, and Glutamate (PASE) sequence (ASPAAPAPASPAEPAPSAPA). The expression vector pET-27b containing the HFt-HIS-PASE gene was assembled by GENEART AG (Thermo Fisher Scientific), considering codon optimisation for high expression levels in *E. coli*. BL21 (DE3) strain (New England BioLabs).

HFt-HIS-PASE was produced with a typical yield of 50mg of pure protein per litre of culture. Cells harbouring the recombinant gene were grown o.n. at 23°C and then at 37°C up to OD600: 1.0, in 1 L of terrific broth medium (Grisp), containing 0.03mg/mL kanamycin (Serva). After induction with 1mM Isopropil-β-D-tiogalattopiranoside (PanReac) bacteria were incubated at 30°C for 2 hours, harvested by centrifugation at 5000 rpm for 20 minutes at 4°C, resuspended in phosphate-buffered saline (PBS) complemented with 150mM NaCl and 20mM imidazole, pH 7.4, protease inhibitors (ThermoFisher Scientific) and 1mM Phenylmethylsulfonyl fluoride (ThermoFisher Scientific). DNAse (0.1 mg/mL final concentration, Sigma-Aldrich) was added before cell disruption by sonication. The lysate was incubated at 37°C for 40 minutes and centrifuged at 14000 rpm for 50 minutes at 4°C. The supernatant was recovered and loaded

in a buffer-equilibrated His-Trap column (Cytiva) for metal affinity chromatography. HFt-HIS-PASE was purified by 100mM imidazole-containing elution buffer, dialysed o.n. against PBS, pH 7.5, and concentrated by 100kDa Amicon Ultra-15 centrifugal filter devices (Millipore). Finally, HFt-HIS-PASE was sterile filtered and stored at 4 °C (short-term) or -20°C (long-term). Preparation purity was assessed by protein staining with Coomassie brilliant blue after a run on 15% SDS-PAGE. Protein concentrations were spectrophotometrically determined at 280 nm, using a molar extinction coefficient (on a 24-mer basis) of 4,56×105 M-1cm-1 (ProtParam sofware, http://www.expasy.org).

HFt-HIS-PASE characterisation

Size-Exclusion Chromatography (SEC) experiments were performed using a Superose 6 gel-filtration column equilibrated with PBS at pH 7,4. All samples were prepared at 1mg/mL in filtered ddH2O. All the traces were analysed with QtiPlot (IONDEV SRL, Bucuresti, Romania).

HFt-HIS-PASE fluorescent labelling

The HFt-HIS-PASE protein (1mg/mL) was incubated with 1mM Fluorescein-5-Maleimide (λex 491 nm, λem 518 nm; ThermoFisher Scientific) in PBS, pH 7 for 2 hours at R.T., under stirring in the dark. The sample was filtered, dialysed, and exchanged with PBS as described above. The sample was sterile filtered and stored at 4 °C in the dark. The number of dyes linked per protein was determined by absorbance spectroscopy, in accordance with the manufacturer's instructions, applying the Lambert–Beer law.

Synthesis of HFt-HIS-PASE-GapmeR complex

GapmeR #1 was encapsulated within the internal cavity of the HFt-HIS-PASE using reversible, pH-dependent cage dissociation (acid pH) and re-association (neutral pH), in the presence of bioactive payload. HFt-HIS-PASE dissociation into subunits was achieved by lowering pH to 2,8 by 0,1M HCl. GapmeR #1 was added to the disassembled protein solution at a molar ratio of 1:1. The pH was then adjusted to 7,4 by 0,1M NaOH, the solution was ultra-filtered at pH 7,4 and exchanged with PBS by 100kDa Amicon Ultra-15 centrifugal filter devices to remove unbound molecules.

Characterisation of HFt-HIS-PASE-GapmeR complex

Electrophoresis on 1,8% agarose gel (AGE) of the purified ferritin-GapmeR complexes was used to demonstrate the loading of GapmeRs in the protein cavity. The gel was run at 90V for 60 min stained with Nucleic Acid SYBR Gold for DNA/RNA visualisation and imaged by ChemiDoc™ (Bio-Rad, America). The amount of GapmeR bound to the ferritin NPs was evaluated by the Image J software (https://imagej.net/ij/), by calculating the area and statistics value of pixels in user-defined selection-objects based on intensity. The same analysis was used to evaluate the concentration of the protein using standards of HFt-HIS-PASE as reference after gel staining with Coomassie dye. Purity and hydrodynamic volume of the HFt-HIS-PASE-GapmeR complexes were determined by SEC experiments. To verify GapmeR encapsulation and protection, HFt-HIS-PASE-GapmeR sample was incubated at 37°C for 1 hour and overnight with 12,5x103 U/mL Denarase (c-LEcta GmbH) to digest DNA and RNA impurities. AGE was used as previously described to determine the GapmeR intensity of each sample. AGE was also employed to check the stability of HFt-HIS-PASE-GapmeR NPs stored at 4 °C for 3 months.

HFt-HIS-PASE-GapmeR delivery

HFt-HIS-PASE-empty and HFt-HIS-PASE-GapmeR complexes were added to D283 Med cell complete growing medium at a concentration of 200nM and maintained for 48 hours. In HFt/DDP double treatment experiments 5µM DDP was added to cells 2 hours after HFt-HIS-PASE administration.

Cytoplasm and nucleus staining

D283 Med cells were cultured as previously described on 0,01% poly-L-ornithine/Murine Laminin 20µg/ml (Sigma Aldrich)-coated coverslips, fixed in 4% paraformaldehyde (Electron Microscopy Sciences) for 20 min at 4 °C and washed with PBS. Fixed cells were then permeabilised and blocked with 0,2% Triton X-100/3% BSA/PBS for 15 min at RT. Subsequently, cells were incubated for 45 min at RT in a solution containing 1% donkey and 1% goat serum in PBS, Alexa Fluor™ 555 Phalloidin 1:100 (A34055, Invitrogen) to mark actin filaments. After washing with PBS, nuclei were counterstained with DAPI solution (1 µg/ml/PBS, D9542, Sigma Aldrich) and the coverslips were mounted using ProLong Diamond Antifade Mountant (Thermo Fisher Scientific, P-36961).

Confocal Microscopy

Samples were imaged using an Olympus iX83 FluoView1200 laser scanning confocal microscope. Acquisitions were performed with a 60X oil objective (NA 1,35) and a 20x objective (NA 0,75) in 16-bit with a resolution of 800 x 800 pixels. For each acquisition with the 60X objective, at least 30 focal planes were imaged with a step size of 0,3µm. For each acquisition with the 20X objective, at least 50 focal planes were imaged with a step size of 0,5µm.

Cell Count Analysis

Cell counting was performed using an Olympus iX83 FluoView1200 laser scanning confocal microscope with a 20X objective as described above. A threshold was set for DAPI (blue, nuclear staining) and for FTH (green, wavelength 488) signals. A maximum intensity projection was created from the Z-stack using Fiji, resulting in a binarised image.

The Fiji "Analyse Particles" tool was employed with a minimum size of 25^2 pixels per field. This approach allowed for the counting of particles in each field for both the green and DAPI signals. The number of green cells was calculated relative to the total number of DAPI-marked cells, providing a percentage of green signal delivery.

Colocalisation Analysis

Colocalisation between signals was analysed using the "JACoP" Fiji plugin. The green signal (wavelength 488 nm), representing FTH, and the red signal (wavelength 555 nm), provided by Phalloidin for cytoplasmic staining, were assessed for colocalisation. Cell nuclei were identified using DAPI (blue). JACoP thresholds of 290 for the green signal and 181 for the blue (DAPI) or red (Phalloidin) signals were applied to all the acquisitions to evaluate the percentage of colocalisation. Manders' coefficients were used to determine the overlap between the signals, with the colocalisation percentages indicating the extent of the signal overlap.

Statistical Analyses

Results are expressed as mean ± SEM from at least 3 biological replicates. In specific cases single experiments were analysed (biological replicates are indicated in figure legends).

Statistical differences between groups were analysed by two-tailed Student's *t*-test (performed with GraphPad QuickCalcs). A *p*-value < 0,05 was considered statistically significant. * $p < 0,05$, ** $p < 0,01$, *** $p < 0,001$, **** $p < 0,0001$. One-way analysis of variance (ANOVA) was applied to multi-group data comparisons, followed by Fisher's LSD test.

Oligonucleotides, probes and GapmeRs

Sequences are listed in DATASET 5
