# Supplementary figures and images for: The MYC-dependent lncRNA MB3 inhibits apoptosis in Group 3 Medulloblastoma by regulating the TGF-β pathway via HMGN5
Source: Cell Death Dis. 2025 Nov 6;16(1):800. doi: 10.1038/s41419-025-08097-8 (PMC12592558; doi:10.1038/s41419-025-08097-8)

Fig 2C

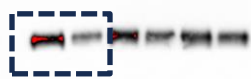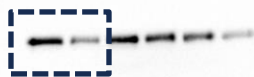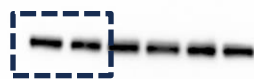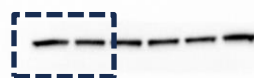

Fig 2D

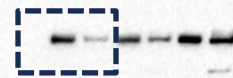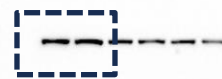

Fig 3B

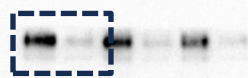

Stripped filter

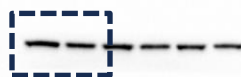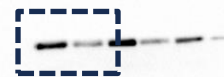

Fig 3D

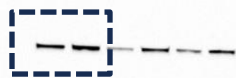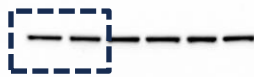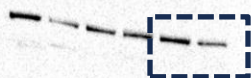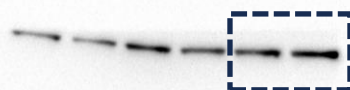

Fig 4G

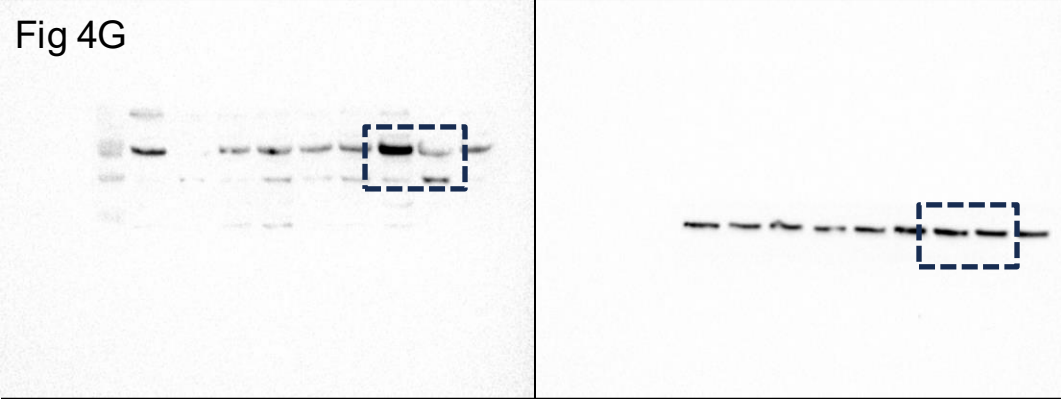

Fig. 4J

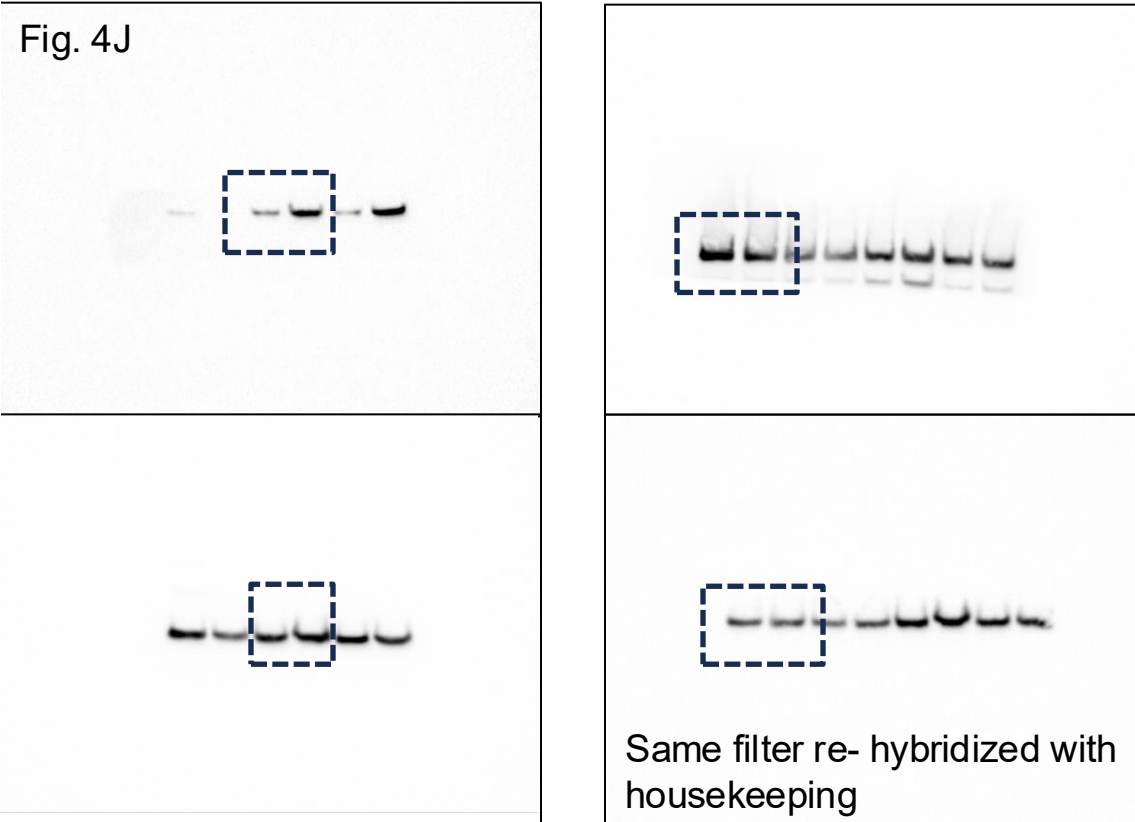

Fig S5A

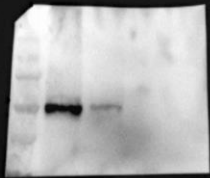

Fig S7A

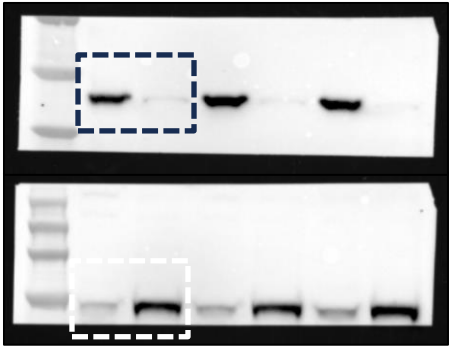

Fig S8B

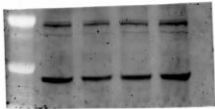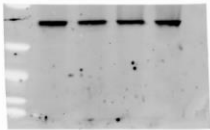

Fig S8C & Fig S8D

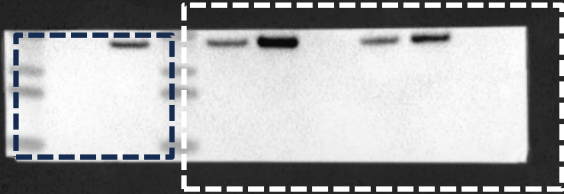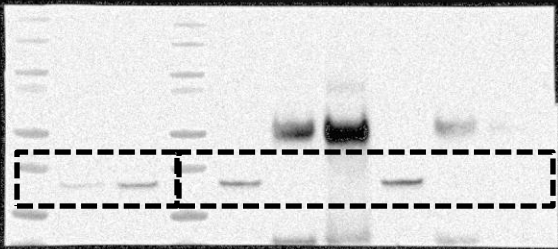

Fig S8F

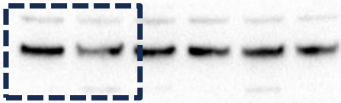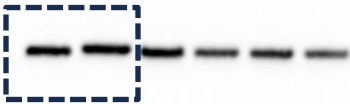

Fig S9C

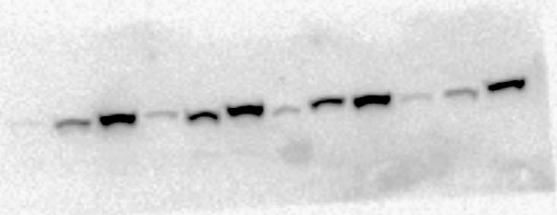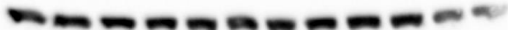

Supplement: Supplementary file 3 — Uncropped wb [file 41419_2025_8097_MOESM3_ESM.pdf]
